# Targeting Mitochondrial Impairment in Parkinson's Disease: Challenges and Opportunities

**DOI:** 10.3389/fcell.2020.615461

**Published:** 2021-01-05

**Authors:** Jannik Prasuhn, Ryan L. Davis, Kishore R. Kumar

**Affiliations:** ^1^Institute of Neurogenetics, University of Lübeck, Lübeck, Germany; ^2^Department of Neurology, University Medical Center Schleswig-Holstein, Lübeck, Germany; ^3^Center for Brain, Behavior, and Metabolism, University of Lübeck, Lübeck, Germany; ^4^Department of Neurogenetics, Kolling Institute, University of Sydney and Northern Sydney Local Health District, Sydney, NSW, Australia; ^5^Department of Neurogenetics, Faculty of Medicine and Health, University of Sydney, Sydney, NSW, Australia; ^6^Molecular Medicine Laboratory and Department of Neurology, Concord Repatriation General Hospital, Faculty of Medicine and Health, University of Sydney, Sydney, NSW, Australia; ^7^Kinghorn Centre for Clinical Genomics, Garvan Institute of Medical Research, Darlinghurst, NSW, Australia

**Keywords:** Parkinson's disease, mitochondria, therapy, mitochondrial dysfunction, neurodegeneration

## Abstract

The underlying pathophysiology of Parkinson's disease is complex, but mitochondrial dysfunction has an established and prominent role. This is supported by an already large and rapidly growing body of evidence showing that the role of mitochondrial (dys)function is central and multifaceted. However, there are clear gaps in knowledge, including the dilemma of explaining why inherited mitochondriopathies do not usually present with parkinsonian symptoms. Many aspects of mitochondrial function are potential therapeutic targets, including reactive oxygen species production, mitophagy, mitochondrial biogenesis, mitochondrial dynamics and trafficking, mitochondrial metal ion homeostasis, sirtuins, and endoplasmic reticulum links with mitochondria. Potential therapeutic strategies may also incorporate exercise, microRNAs, mitochondrial transplantation, stem cell therapies, and photobiomodulation. Despite multiple studies adopting numerous treatment strategies, clinical trials to date have generally failed to show benefit. To overcome this hurdle, more accurate biomarkers of mitochondrial dysfunction are required to detect subtle beneficial effects. Furthermore, selecting study participants early in the disease course, studying them for suitable durations, and stratifying them according to genetic and neuroimaging findings may increase the likelihood of successful clinical trials. Moreover, treatments involving combined approaches will likely better address the complexity of mitochondrial dysfunction in Parkinson's disease. Therefore, selecting the right patients, at the right time, and using targeted combination treatments, may offer the best chance for development of an effective novel therapy targeting mitochondrial dysfunction in Parkinson's disease.

## Introduction

Parkinson's disease (PD) is a common disorder, with over 6 million individuals affected globally (Collaborators, 2019). With only symptomatic treatments available, the greatest current challenge is to develop an effective disease-modifying therapy based on an understanding of the underlying disease mechanisms. While the pathophysiology of PD is complex, mitochondrial dysfunction has an established central role. A growing body of research has provided insights into the diversity of mechanisms governing mitochondrial dysfunction in PD. Thus, targeting mitochondrial dysfunction is a promising approach for the development of future therapies. Numerous trials focused on ameliorating mitochondrial dysfunction in PD have been conducted but overall have been unsuccessful to date.

We will review previous efforts, the development of new therapies and potential targets yet to be exploited for mitochondrial dysfunction in PD. We highlight the importance of targeting the right patients at the right time for clinical trials, and the need for relevant and accurate biomarkers to monitor response to therapy. We discuss how a combined approach targeting various aspects of mitochondrial dysfunction may serve as the best pathway forward to developing an effective treatment in PD.

### Genetic Studies of PD Have Provided Insights Into Disease Pathophysiology

PD is a multifactorial disease with a diverse genetic, biological, and environmental background. The majority of PD is thought to be non-Mendelian and is termed “idiopathic PD” (IPD). The pathophysiology underlying the vast majority of IPD patients is complex and to date, only partially understood (Blauwendraat et al., 2020). Monogenic forms of PD (mPD) have been linked to multiple cellular pathways, the investigation of which has improved our understanding of the molecular basis of IPD in a reductionist manner (Supplementary Table 1). Many of the identified forms of mPD have been linked to impaired mitochondrial homeostasis, including pathways that are also relevant for IPD (Larsen et al., 2018). Furthermore, genome-wide association studies have identified IPD risk loci within mPD genes. Many mPD genes and IPD risk loci have proven relationships to mitochondrial dysfunction. For example, *PRKN, PINK1*, and *LRRK2*-related cases show distinct disturbances in mitochondrial-related pathways (Bose and Beal, 2016). *DJ-1*-related PD may relate to a disturbance of the cytoprotective role against oxidative stress with a plausible link to mitochondrial dysfunction, despite a lack of detail on mechanistic involvement (Dolgacheva et al., 2019). It is essential to recognise that these pathways often influence one another and that each of the PD-linked gene products, when dysfunctional, can impact multiple pathways either directly or indirectly. When considering the centrality of mitochondria in cellular and metabolic homeostasis and the predominance of mitochondrial dysfunction in PD pathogenesis, it would appear that a tapestry of interconnected pathways and events, rather than a single pathogenic pathway, is the conceptual hurdle that must be addressed by therapy. This may, in part, explain why the neuroprotective strategies trialled to date that are directed at a single target have generally been unsuccessful at producing clear benefits (Supplementary Table 2).

### Mitochondrial Dysfunction Plays a Central and Multifaceted Role in Parkinson's Disease

**Figure 1:**
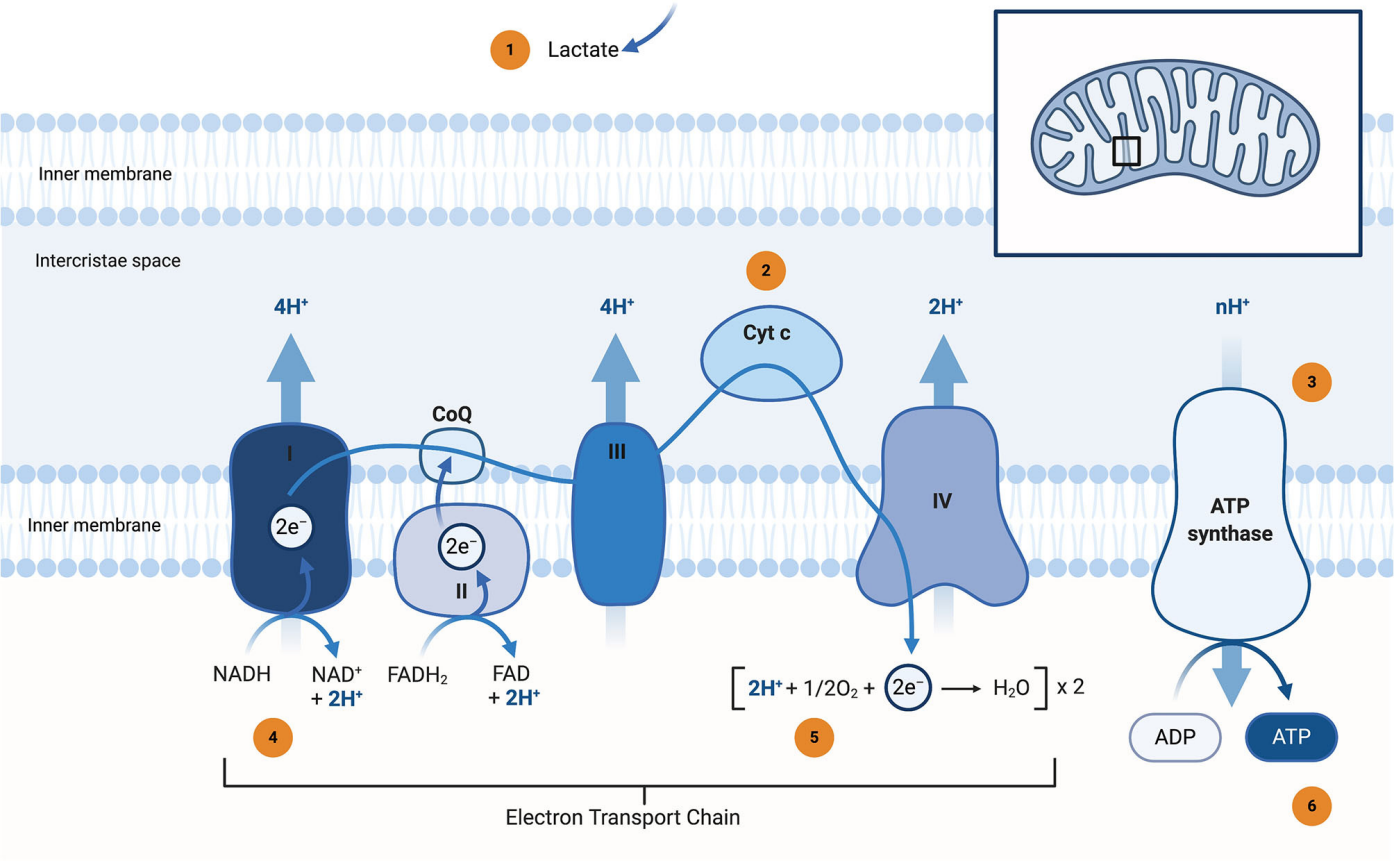
Schematic representation of the Electron Transport Chain (ETC) and potential neuroimaging modalities to assess bioenergetic disturbances in Parkinson's disease (PD) patients. The localisation of the ETC complexes is highlighted in the right upper corner. Lactate (as a surrogate marker for mitochondrial dysfunction) can be assessed by ^1^H-MRSI **(1)**. bNIRS devices can measure Cyt c oxidation state under dynamic conditions **(2)**. ADP and ATP can either be measured under dynamic (ATP synthase activity) by ^31^P-MRSI magnetisation transfer **(3)** or static conditions (regular ^31^P-MRSI) **(6)**. Complex I activity can be studied by ^31^P-MRSI (by measuring the NAD+/NADH ratio) or via a radiolabeled PET tracer (^18^F-BCPP-EF) **(4)**. Many different approaches can be used to study *in-vivo* oximetry and oxygen turnover. The most common non-invasive methods are ^17^O-MRI (direct measurement) or a combination of BOLD and ASL imaging (indirect measurement by employing different physiological models) **(5)**. ^1^H-MRSI, proton magnetic resonance spectroscopy imaging; ^17^O-MRI, oxygen magnetic resonance imaging; ^18^F-BCPP-EF, 2-tert-butyl-4-chloro-5-[6-[2-2[([^18^F]fluoroethoxy)-ethoxy]-pyridin-3-ylmethoxy]-2H]-pyridazin-3-one; ^31^P-MRSI, phosphorus magnetic resonance spectroscopy imaging; ADP, adenosine diphosphate; ASL, arterial spin labelling; ATP, adenosine triphosphate; bNIRS, broadband near-infrared spectroscopy; BOLD, blood oxygen-dependent imaging, functional MRI; CoQ, coenzyme Q; Cyt c. Cytochrome c; e^−^, electron; FAD, flavin adenine dinucleotide (oxidised form); FADH_2_, flavin adenine dinucleotide (reduced form); H^+^, proton; H_2_O, water; I, complex I; NADH, ubiquinone oxidoreductase, Type I NADH dehydrogenase; II, complex II, Succinate dehydrogenase, succinate-coenzyme Q reductase; III, complex III, coenzyme Q: cytochrome c—oxidoreductase; IV, complex IV, cytochrome c oxidase; MRI, magnetic resonance imaging; *n*, number; NAD^+^, nicotinamide dinucleotide (oxidised form); NADH, nicotinamide dinucleotide (reduced form); O_2_, molecular oxygen; PD, Parkinson's disease; PET, positron emission tomography. *Created with* *https://biorender.com/*.

Mitochondrial dysfunction plays a fundamental and complex role in many neurodegenerative disorders, including PD (Grimm and Eckert, 2017). PD-associated mitochondrial dysfunction can result from a number of causes, including impairment of mitochondrial biogenesis, increased reactive oxygen species (ROS) production, defective mitophagy, compromised trafficking, electron transport chain (ETC) dysfunction (Figure 1), variations to mitochondrial dynamics, calcium (Ca^2+^) imbalance and possibly other indirect influences on mitochondrial function from unrelated pathways (Park et al., 2018; Grunewald et al., 2019). If these insults cannot be overcome by protective mechanisms, a relentless cycle of dysfunction will eventually evoke all these dysfunctions, leading to cellular impairment and ultimately cell death. Besides the mitochondrion's major function of generating cellular energy in the form of adenosine triphosphate (ATP), their involvement in the regulation of cell death via apoptosis, Ca^2+^ homeostasis, haem biosynthesis, and the formation and export of iron-sulphur (Fe-S) clusters, control of cell division, and growth have also been shown to be altered in IPD and mPD (Bose and Beal, 2016).

During the oxidative phosphorylation process, electrons can leak from the ETC, largely from complex I (C.I) and complex III (C.III), and react with molecular oxygen to form superoxide (${\text{O}}_{2}^{-}$), one of the reactive oxygen species (ROS) (Rani and Mondal, 2020). Under physiological conditions, this production occurs at relatively low levels and is removed by mitochondrial antioxidants, such as manganese superoxide dismutase (MnSOD or SOD2), glutathione (GSH) and the peroxiredoxins. MnSOD converts ${\text{O}}_{2}^{-}$ to hydrogen peroxide (H_2_O_2_), which is then converted to H_2_O by GSH, as part of the network that removes H_2_O_2_ (Mischley et al., 2016). A reduction in the concentration of GSH in the substantia nigra pars compacta (SNpc) could be an early and modifiable event for PD. The provision of energy by oxidative phosphorylation is probably the most striking feature of mitochondria, and respective disturbances are common in mPD and IPD. Furthermore, environmentally-induced PD can occur as a result of compounds that inhibit the mitochondrial ETC, such as rotenone (Ramalingam et al., 2019). As most drug candidates target the ETC (usually by bypassing defective ETC complexes), the question arises whether this approach neglects the complexity and widespread function of this fundamental cell organelle. Mitochondria are composed of a double lipid bilayer with a permeable phospholipid outer membrane and an impermeable phospholipid inner membrane that surrounds the intra-compartmental matrix. This compartmentalization leads to additional pharmacodynamic challenges in drug delivery (Murphy and Hartley, 2018).

### The Cause and Consequence Dilemma of Mitochondrial Dysfunction in Parkinson's Disease Aetiology

Mitochondrial dyshomeostasis is a significant factor in PD neuronal cell death (Liu et al., 2018). Since the initial discovery of C.I dysfunction in PD, our understanding of the complexity and interconnectedness of mitochondria in PD's pathogenesis has considerably expanded. The overall high prevalence of mitochondrial dysfunction in PD is likely underpinned by the fundamental role of mitochondria in cellular energy production, as well as their role in the majority of metabolic pathways, their centrality to cellular homeostasis, and their overall mediation of cell survival (Angelova and Abramov, 2018). Despite the overwhelming evidence for the pathophysiological role of mitochondria in PD and their pivotal role in diverse cellular pathways, the disentanglement of mitochondrial dysfunction as a causative or consequential factor and the identification of the seminal events leading to neurodegeneration remains challenging (Park et al., 2018).

Interestingly, inherited mitochondriopathies do not usually present with parkinsonian features [with some notable exceptions, such as in *POLG* mutation carriers (Ma et al., 2020)]. The primary evidence for a causative role of mitochondrial dysfunction are studies on mPD and environmental studies. In addition, phenotypes consistent with IPD can be induced by several endogenous and exogenous inhibitors of mitochondrial function, including rotenone, 1-methyl-4-phenyl-1,2,3,6-tetrahydro-pyridine (MPTP), paraquat, nitric oxide, and the dopamine metabolite aminochrome (Bellou et al., 2016). MPTP is a potent inhibitor of C.I in the ETC, yet paradoxically, parkinsonism is generally not present in patients with genetic C.I deficiency (Langston, 2017). Therefore, the extent to which mitochondrial dysfunction alone can be a precipitating factor in PD is still uncertain.

It is becoming increasingly apparent that the reductionist approach to mitochondrial dysfunction (by focusing solely on the ETC and its production of ATP) oversimplifies their complex role in controlling cellular homeostasis. However, the complexity could also provide an opportunity for drug development, as many cellular alterations in PD (and other neurodegenerative disease for that matter) can be modulated by alleviating mitochondrial dysfunction. Genetic PD may be both primarily mitochondrially involved but also indirectly involved; therefore, targeting only the mitochondria will be insufficient as a stand-alone therapeutic approach. This may particularly be the case if mitochondrial dysfunction is the consequence rather than the cause of disease pathophysiology (e.g., in *ATP13A2* mutation carriers with Kufor-Rakeb Syndrome; Park et al., 2014, 2016; Wang Z. B. et al., 2019).

### Recognising Prodromal Mitochondrial Dysfunction: Selection of Study Participants and the Role of Neuroimaging

The temporal dynamics of mitochondrial dysfunction in PD are unclear. For example, there is currently no experimental evidence to determine whether ROS production leads to dysfunctional ETC complexes or dysfunctional ETC complexes cause increased production of ROS (Singh et al., 2019). But the temporal aspects of mitochondrial dysfunction are crucial for understanding PD pathophysiology and accelerating future drug development. Identifying at-risk individuals and treating them in a prodromal phase based on their driving pathophysiological process could prevent the manifestation of PD, or at least alter the disease progression (Ascherio and Schwarzschild, 2016).

At present, two approaches could be feasible to investigate this matter: the a-priori stratification of study cohorts (based on assumed mitochondrial dysfunction) or the *in-vivo* assessment of mitochondrial dysfunction (by neuroimaging methods or blood biomarkers) (Prasuhn et al., 2019). It is desirable to enrich study cohorts with participants exhibiting mitochondrial impairment, to ensure the success of clinical trials directly targeting the mitochondria. Enrichment or stratification of study cohorts by mPD (e.g., *PRKN* mutation carriers) fosters this approach. However, the extension of potential findings to the vast majority of IPD patients remains challenging. Polygenic risk scoring of *in-silico* annotated single nucleotide polymorphisms (SNPs) could translate the concept of mitochondrial impairment found in monogenic PD to IPD (Prasuhn et al., 2019). However, for most useful SNPs, functional studies are lacking and require validation for humans in advance of clinical trials.

**Figure 2:**
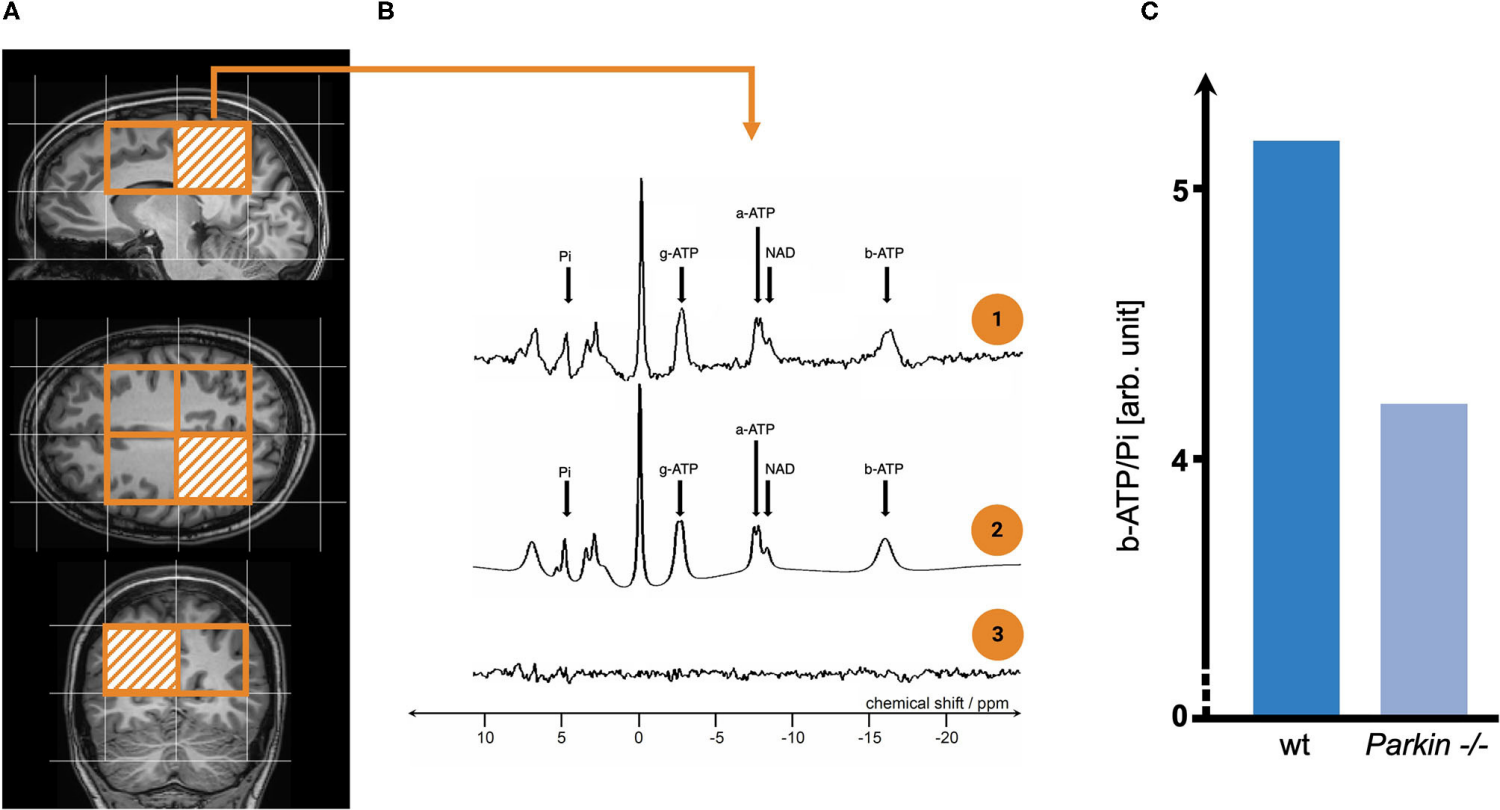
The exemplary workflow of phosphorus-magnetic resonance spectroscopy imaging (^31^P-MRSI) analysis and its potential role in imaging mitochondrial dysfunction in Parkinson's disease (PD) patients. **(A)** Depicts the voxel placement (orange bordered) for metabolic studies *in-vivo*. As seen here, one of the most significant limitations in ^31^P-MRSI studies is the relatively poor spatial resolution. The arbitrarily chosen voxel-of-interest is highlighted by orange striation. We receive a spectrum of phosphorus-containing metabolites for each voxel (see **B.1**), which we marked by small black arrows. a/b/g-ATP do not reflect the number of covalently bound phosphate groups (by means of AMP/ADP, and ATP) but result, e.g., from electron-mediated interaction of two (or more) nuclear spins residing on the same molecule (among other quantum physical mechanisms). Usually, the raw spectrum (see **B.1**) is processed by mathematical operations that incorporate prior knowledge on investigated molecules' physical properties (see **B.2**). In **(B.3)**, one can see the 'unfitted' noise of the raw spectrum. To illustrate how ^31^P-MRSI can be applied to study mitochondrial dysfunction in PD, two exemplary patients were investigated here (see **C**): One patient carrying two wild type (wt) alleles for the *Parkin* allele and, in contrast, one patient carrying two homozygous loss-of-function mutations (*Parkin*^−/−^). The spectral integral of investigated metabolites (here b-ATP and Pi) is used for the (semi-)quantification of metabolite content. Commonly, metabolites are normalised (as a ratio to Pi) to account for the overall phosphorus amount in human brain tissue (which might be influenced by, e.g., nutritional intake). -/-, homozygous *Parkin* mutation carrier; ^31^P-MRSI, phosphorus magnetic resonance spectroscopy imaging; a/b/g-ATP, alpha/beta/gamma-adenosine triphosphate; ADP, adenosine diphosphate; AMP, adenosine monophosphate; arb. unit, arbitrary unit; NAD, nicotinamide adenine dinucleotide; PD, Parkinson's disease; Pi, inorganic phosphate; ppm, parts per million; wt, wild type.

Secondly, the *in-vivo* assessment of key aspects of mitochondrial dysfunction via specific neuroimaging methods is likely to be highly advantageous. Magnetic resonance spectroscopy imaging (MRSI) could provide a non-invasive approach to assess mitochondrial bioenergetics. ^31^Phosphorus-MRSI (^31^P-MRSI) bears the potential to measure ATP among other phosphorus-containing metabolites (Forester et al., 2010; Figure 2). ^31^P-MRSI has been used to measure the ratio of NAD^+^/NADH, which offers unique opportunities for recent niacin-based clinical trials in PD [(Lehmann et al., 2017), REPAIR-PD: NCT03815916]. In addition, proton-MRSI (^1^H-MRSI) can be applied to measure lactate levels as a surrogate marker of impaired oxidative phosphorylation (Henchcliffe et al., 2008).

Continuous methodological improvements of scanner hardware (by increasing the static magnetic field strengths) or MRI sequence optimization, offer great potential for MRSI studies (Henchcliffe et al., 2008). ^1^H-MRSI can be used to measure glutathione levels, which helps assess the oxidative state of PD brain tissue (Mischley et al., 2016). Further proof is required to determine whether the latter approach might benefit studies investigating the potential therapeutic use of glutathione or other antioxidants in PD. However, a recent placebo-controlled and randomised clinical trial with intranasal administration of glutathione in unselected PD patients did not show superiority to placebo using the Unified PD Rating Scale as an endpoint (Mischley et al., 2017). Promisingly, *in-vivo* oximetry (as a combination of different MRI modalities, such as arterial spin labelling and BOLD imaging, susceptibility-weighted imaging, or ^17^O-MRI) shows potential for discerning *in situ* mitochondrial impairment (Borghammer et al., 2008).

Besides MRI-based methods, broadband near-infrared spectroscopy (bNIRS) can be employed to study the oxidation status of cytochrome c by multiwavelength absorption and reflection of cortical cytochrome c in the near-infrared spectrum of light (Lange et al., 2019). Unfortunately, bNIRS devices are currently not commercially available, with limited application to date. Additionally, positron emission tomography (PET) provides additional insights into mitochondrial dysfunction, with a novel PET probe, ^18^F-BCPP-EF, recently used to examine C.I activity in PD patients (Wilson et al., 2020).

Neuroimaging studies on mitochondrial impairment in PD are still scarce and lack intra-site reliability (e.g., by distinct hardware requirements), which is one major prerequisite for theranostic-accompanied drug trials (Dossi et al., 2019). The ongoing development of quantitative MRI/MRSI sequences and dual-calibrated fMRI (for MRI-based oximetry) will more than likely overcome the problem of inter-site reliability (Lara et al., 1993; Germuska et al., 2019). However, current neuroimaging studies often lack reference values to interpret findings, and longitudinal studies are needed to identify bioenergetic disturbances' temporal dynamics. Aside from the unknown temporal dynamics of mitochondrial dysfunction, clinical trials in neurodegenerative diseases, in general, face unique challenges with long interventional periods, the need for sophisticated trial designs (delayed-clinical trials or adaptive designs), and a priori patient-stratification. At present, the proposed neuroimaging methodology only addresses alterations in mitochondrial bioenergetics and neglects other (maybe earlier) pathophysiological hallmarks. Future studies in this area are urgently needed and will play a pivotal role in future clinical trials' success.

### Blood Biomarkers of Mitochondrial Dysfunction

In addition to neuroimaging findings, blood-based biomarkers may also be helpful to assess mitochondrial dysfunction *in vivo*. One general concern of blood-based biomarkers is that they do not recapitulate mitochondrial dysfunction of neuronal cell populations. Gene mutations leading to mPD often show tissue-specific expression patterns and therefore lack biological interpretability in peripheral blood cells, e.g., peripheral monoclonal blood cells (PMBCs; Dossi et al., 2019; He et al., 2019). The determination of mtDNA mutation load is altered by the different proliferation rates of distinct cell types, which hinders the insights gathered from PMBCs to assess neuronal dysfunction (O'Callaghan et al., 2015). This mainly applies to most of the current approaches in measuring mitochondrial dysfunction via the measurement of mitochondrial membrane potential, C.I activity, among other functional assays. Besides, it is still unclear which neuronal cell types are affected by mitochondrial dysfunction. As most (pre-)clinical studies investigate the influence of mitochondrial dysfunction on dopaminergic neurons, other neuronal cell types and neurotransmitter systems are likewise involved in PD pathogenesis. The use of mitochondria-specific blood biomarkers would most likely not assess the selective loss of dopaminergic neurons, comparing the small size of the substantia nigra to the overall large human blood volume. Supporting evidence comes from a study comparing the amounts of FGF-21 and GDF-15 (as well-established biomarkers of mitochondrial disease), showing negligible diagnostic value in assessing mitochondrial dysfunction of PD patients (Davis et al., 2020).

## Therapeutic Approaches

### Recovering Physiological ATP Production by Targeting the ETC and Antioxidative Treatment Strategies

Based on the close interconnectedness of ETC complex dysfunction and increased oxidative stress, most compounds trialled to date essentially influence both aspects of mitochondrial dyshomeostasis. Coenzyme Q10 (ubiquinone; CoQ10) and its derivatives are still the most studied investigational products (Negida et al., 2016; Zhu et al., 2017). The proposed method of action of CoQ10 is to bypass dysfunctional C.I by electron transfer via the Q-cycle (Yang et al., 2016). Also, CoQ10 could directly decrease the extent of oxidative stress (Yang et al., 2016). Although studies in animal models yielded promising results, most clinical trials failed to reach relevant clinical endpoints (Supplementary Table 2), possibly because of the unselected nature of the PD patients enrolled (Negida et al., 2016; Zhu et al., 2017; Attia and Maklad, 2018). To overcome additional pharmacodynamic and kinetic challenges, different galenic formulations (e.g., nano-emulsified ubiquinol) or derivatives of CoQ10 (e.g., EPI 589) were developed (Kumar et al., 2016; NCT02462603). Vitamin K2 (long-chain menaquinone 7; MK-7) may also act like CoQ10 based on their structural similarity, as demonstrated by studies in *Drosophila* flies carrying a homozygous *PINK1* knockout (Vos et al., 2012). However, these findings need to be validated in human trials.

Nicotinamide (vitamin B3, NAM) and its derivatives are also currently under investigation, where the underlying rationale is to normalise redox levels (NAM may also affect sirtuins, as discussed later). NAM, particularly NAD+ and NADH, are also highly relevant for C.I and could be beneficial for ETC disturbances (Lehmann et al., 2017; REPAIR-PD: NCT03815916). The innovative REPAIR-PD study (phase 2) assesses the cerebral metabolic effects, safety, pharmacokinetics, and pharmacodynamics of an oral, gold nanocrystal liquid suspension (CNM-Au8, Supplementary Table 2). Proposed antioxidative properties will be investigated by measuring the *in-vivo* NAD^+^/NADH ratio via ^31^P-MRSI.

N-acetyl-cysteine (NAC) has also been investigated in a small proof-of-concept study. It showed antioxidative properties measured by increased blood and brain glutathione levels after single-time point administration (Monti et al., 2019). The natural compound apocynin (Apo) has mitigating properties that are involved in inflammatory responses (Cheng et al., 2018). Apo has been adjusted to target mitochondria (Mito-Apo) (Ghosh et al., 2016; Langley et al., 2017), with preclinical PD models showing that it could prevent MPTP-induced nigral cell loss, indicating its potential use for mitochondrial dysfunction in PD (Ghosh et al., 2016; Langley et al., 2017). Another investigational drug candidate that has been assessed in animal models is N-Methyl, N-propynyl-2-phenylethylamine (MPPE). MPPE serves as an MAO-B inhibitor that prevents MPTP-induced nigral cell loss, upregulates mitochondrial superoxide dismutase to alleviate oxidative stress, and improves C.I function (Shin et al., 2016). Additionally, the S(-) enantiomer of pramipexole, an often used dopamine agonist in routine clinical care, has also been shown to have antioxidative properties (Izumi et al., 2007). Unfortunately, it seems to lack disease-modifying efficacy in humans.

The most recent clinical trial candidate showing promise is ursodeoxycholic acid (UDCA), a drug often used in chronic inflammatory liver disease with an extensive safety profile (Sathe et al., 2020). UDCA has been shown to prevent mitochondrial membrane depolarisation and stabilises cytochrome c in the mitochondrial membrane (Abdelkader et al., 2016; Bell et al., 2018). There is also convincing *in vivo* evidence that UDCA can be especially beneficial for treating mitochondrial impairment in *LRRK2*^*G*2019*S*^ mutation carriers (Mortiboys et al., 2015). An upcoming oral UDCA study is also coupled with the study of brain energy metabolism via ^1^H-MRSI (NCT02967250).

### Exercise

The effect of physical exercise has been assessed in both animal models and PD patients and is shown to promote mitochondrial biogenesis and function.

The long-term effects of voluntary exercise were investigated in a transgenic MitoPark mouse PD model (Lai et al., 2019). Voluntary exercise was found to improve behavioural parameters and nigrostriatal dopamine input. Additionally, exercise increased oxygen consumption, in keeping with increased ATP production via oxidative phosphorylation (Lai et al., 2019).

In a unilateral PD rat model, rats were subjected to either 1 or 4 weeks intermittent moderate treadmill exercise (Fernandes Ferreira et al., 2020). The investigators showed that 1 week of exercise prevented a decrease in peroxisome proliferator-activated receptor gamma coactivator-1 alpha (PGC-1α) and NRF-1 expression. Furthermore, 4 weeks of exercise prevented a reduction in transcription factor A, mitochondrial (TFAM) and C.I protein levels and augmented C.I activity. This suggests that intermittent exercise improves mitochondrial biogenesis signalling and respiratory chain modulation of the dopaminergic system in PD (Fernandes Ferreira et al., 2020).

Another study using a rodent model showed that exposure to 6-hydroxydopamine (6-OHDA) led to a reduction of mitochondrial factors adenosine monophosphate (AMP)-activated protein kinase, PGC-1α, and tyrosine hydroxylase (TH), and increased expression of silent information regulator T1, TFAM, and *p53* (Rezaee et al., 2019). Notably, gene and protein expressions upon exercise were elevated and the *p53* protein levels were lower in an exercise and 6-OHDA group compared with a no exercise and 6-OHDA group.

Furthermore, endurance exercise restored motor function and reduced apoptosis in a MPTP mouse model (Jang et al., 2018). These benefits were associated with mitochondrial phenotypic changes such as upregulated anti-apoptotic proteins, reduced pro-apoptotic proteins and improved mitochondrial biogenesis and fusion (Jang et al., 2018).

In addition to rodent PD models, *Drosophila Parkin* mutants were used to demonstrate that dietary management along with physical activity has the potential to improve mitochondrial biogenesis and delay the progression of PD (Bajracharya and Ballard, 2018).

A recent study in humans showed that exercise-induced improvements in the PD clinical state were associated with specific adaptive changes in muscle functional, metabolic, and molecular characteristics, although some parameters, such as muscle mitochondrial DNA content, improved with exercise in controls and not in PD patients (Krumpolec et al., 2017).

There are many advantages to exercise; it is inexpensive, practical, sustainable, and has additional health benefits (Lai et al., 2019). Overall, the evidence supports an unambiguous benefit of exercise in PD, which is at least partly explained by a beneficial effect upon mitochondrial function.

### Enhancing the Clearance of Dysfunctional Mitochondria via Mitophagy or Other Mitochondrial Stress Response Pathways

Multiple lines of evidence point to the importance of mitophagy in the pathophysiology of PD. For example, the *PRKN* and *PINK1* genes mediate mitophagy (Pickrell and Youle, 2015) and are the major causes of autosomal recessive early onset mPD (Supplementary Table 1). Therefore, enhancing mitophagy is a key therapeutic strategy in PD (Aman et al., 2020). In keeping with this, investigators used a rodent model of PD to study the effect of kinetin, the precursor of kinetin triphosphate, an activator of both wild-type and mutant forms of *PINK1* (Orr et al., 2017). However, in *PINK1* null rodents, no degeneration of midbrain dopamine neurons was identified. Additionally, in rodent models of α-synuclein induced toxicity, boosting PINK1 activity with oral kinetin provided no protective effects, thus showing no evidence of a beneficial effect in a preclinical model of IPD (Orr et al., 2017). Another agent, celastrol, was shown to exert neuroprotective effects through activating mitophagy and inhibiting dopaminergic neuronal loss in PD cell and mouse models (Lin et al., 2019). Recently, a study used a high-throughput phenotype detection system for drug screening in dopaminergic neurons from induced-pluripotent stem cells (iPSCs) derived from patients with PD due to *PRKN* or *PINK1* mutations (Yamaguchi et al., 2020). After screening 320 compounds, they identified 4 candidate drugs that were effective for ameliorating impaired mitochondrial clearance, showing the utility of this method for identifying candidate PD drugs (Yamaguchi et al., 2020).

### Improving Mitochondrial Biogenesis

Mitochondrial biogenesis is a complex process involving coordination of transcription, translation, import of nuclear-encoded components, as well as the expression of mitochondrial genes (Chandra et al., 2019). A recent study of Parkin-deficient human dopaminergic neurons demonstrated that while there was defective mitophagy in human dopaminergic neurons lacking Parkin, the mitochondrial dysfunction is chiefly a consequence of defects in mitochondrial biogenesis (Kumar et al., 2020). The defective mitochondrial biogenesis is driven by the upregulation of the Parkin substrate PARIS and the subsequent downregulation of PGC-1α (Ge et al., 2020; Kumar et al., 2020). Thus, strategies aimed at enhancing mitochondrial biogenesis should be a focus for the development of new therapeutic approaches to treat PD (Kumar et al., 2020).

Targeting of the AMP-activated protein kinase (AMPK)-SIRT1-PGC-1α axis may be the most promising approach to enhancing mitochondrial biogenesis, and could involve PGC-1α activating drugs targeting PPAR, AMPK, and SIRT1 (Chandra et al., 2019). A study using a 6-OHDA lesioned rat model of PD showed that the polyphenolic phytochemical ferulic acid can modulate PGC1α with beneficial effects on mitochondrial dynamics, supporting the concept of targeting PGC1α, a master regulator of mitochondrial biogenesis, as a therapeutic strategy (Anis et al., 2020; Bennett and Keeney, 2020). A recent study demonstrated a novel approach to increasing mitochondrial biogenesis in neuronal cells via RNS60 (0.9% saline solution containing oxygenated nanobubbles), through phosphatidylinositol 3-kinase-mediated upregulation of PGC1α (Chandra et al., 2018, 2019). Moreover, the drug exenatide has been shown to improve motor scores in PD and may have beneficial effects on mitochondrial biogenesis (Fan et al., 2010; Athauda et al., 2017). Additionally, dopamine D1 receptor agonism has been found to improve mitochondrial biogenesis and dopaminergic neurogenesis in a 6-OHDA rat model of PD (Mishra et al., 2020). Finally, baicalein, a bioactive flavone of *Scutellaria baicalensis Georgi*, has been shown to enhance mitochondrial biogenesis in a rotenone-induced PD rat model (Zhang et al., 2017).

### Gene Therapies Targeting Mitochondrial Dysfunction in Parkinson's Disease

Gene therapy entails the treatment of disease via delivery of a transgene that either replaces or corrects a defective gene or is generally supportive of cells in the disease environment (O'Connor and Boulis, 2015). Gene therapy vectors can be either viral [commonly adeno-associated viruses (AAVs) and lentiviruses (LVs)] or non-viral (typically naked plasmid DNA or in complex with cationic lipids or polymers) (O'Connor and Boulis, 2015).

Autosomal recessive mutations in the *PRKN* gene cause loss of function (truncating variants) or inactivation (missense variants) of the parkin protein (Bruggemann and Klein, 1993). Overexpression of *PRKN* has been shown to have a protective effect against numerous cellular insults (Choong and Mochizuki, 2017). For example, overexpression of wild type parkin in a transgenic mouse model was shown to alleviate MPTP-induced dopaminergic neurodegeneration via protection of mitochondria and decreased striatal alpha-synuclein (Bian et al., 2012). Furthermore, recombinant AAV vector-mediated intranigral delivery of parkin prevented motor deficits and dopaminergic cell loss in a chronic MPTP-minipump mouse model of PD (Yasuda et al., 2011).

Loss of function mutations in *PINK1* can also lead to autosomal recessive PD (Valente et al., 2004). Removal of *Drosophila PINK1* homologue function leads to male sterility, apoptotic muscle degeneration, defects in mitochondrial morphology and increased sensitivity to multiple stressors including oxidative stress. In a portion of PINK1 mutants, expression of human PINK1 in the Drosophila testes restores male fertility and normal mitochondrial morphology (Clark et al., 2006).

Numerous studies indicate that mitochondrial pathology and muscle and dopaminergic degeneration due to *Drosophila* PINK1 inactivation and *PINK1* mutant can be rescued by *PRKN* overexpression and downregulation of Miro (Yang et al., 2006; Liu and Lu, 2010; Liu et al., 2012; Choong and Mochizuki, 2017). Furthermore, overexpression of *PINK1* results in the rescue of the alpha-synuclein-induced phenotype in a *Drosophila* model of PD (Todd and Staveley, 2008). Depletion of PINK1 by RNA interference increased neuronal toxicity induced by 1-Methyl-4-phenylpyridinium ion (MPP^+^) (Haque et al., 2008). Moreover, wild-type *PINK1*, but not the mutant form, protects neurons against MPTP/MPP^+^ both *in vitro* and *in vivo* (Haque et al., 2008). Furthermore, viral-mediated expression of *PRKN* and *DJ-1* genes can protect dopaminergic neurons, even in the absence of PINK1, suggesting that DJ-1 and Parkin act in parallel or downstream of endogenous PINK1 to mediate survival (Haque et al., 2012).

In general, studies of autosomal recessive genes, such as *PRKN* and *PINK1*, support a loss of function mechanism ameliorated by replacement, thus suggesting gene therapy as a promising treatment strategy in PD. In addition, gene therapy based on transfection of mtDNA-complexed *TFAM* or recombinant *TFAM* to PD cybrid cells shows potential to restore mitochondrial bioenergetics of severely impaired nigral neurons, as TFAM is important for mtDNA maintenance (Keeney et al., 2009; Golpich et al., 2017).

Somatic mtDNA point mutations may be elevated in the substantia nigra of patients with PD (Lin et al., 2012). A recent study used bacterial cytidine deaminase toxin for CRISPR (Clustered Regularly Interspaced Short Palindromic Repeats)-free mitochondrial base editing, allowing for the prospect of correcting mitochondrial mutations (Mok et al., 2020), a technique which may find utility for PD in the future.

### Protein-Based Therapies Targeting Mitochondrial Dysfunction in Parkinson's Disease

A protein-based approach may be utilised to address mitochondrial dysfunction in PD. Cell-permeable Parkin protein (iCP-Parkin) was recently investigated as a protein-based therapy in cellular and animal-based models (Chung et al., 2020). iCP-Parkin promoted mitophagy and mitochondrial biogenesis, thereby recovering damaged mitochondria, suppressing toxic accumulation of alpha-synuclein, and preventing and reversing declines in TH and dopamine expression and improving motor function. These findings support iCP-Parkin as a potential PD-modifying agent (Chung et al., 2020).

### Nrf2/ARE as a Drug Target for Parkinson's Disease

The nuclear factor erythroid 2-related factor 2 (Nrf2)-antioxidant response element (ARE) signalling cascade plays a key role in several aspects of mitochondrial homeostasis, such as mitochondrial biogenesis, mitophagy, ROS production and scavenging (Gureev and Popov, 2019). This makes the Nrf/ARE pathway a highly appealing target for a novel drug therapy for PD, which may be used in tandem with antioxidant protection to slow disease progression (Gureev and Popov, 2019). Very recently, an activator of the Nrf2-ARE pathway, TPNA10168, was investigated in a PD rodent model (Inose et al., 2020). TPNA10168 was found to inhibit dopaminergic neuronal death, and it was thought that heme oxygenase-1, an antioxidant enzyme expressed downstream of the Nrf2-ARE signalling pathway, might participate in this effect.

### Restoring Mitochondrial Dynamics and Trafficking

Mitochondria form a complex network, the cohesiveness and shape of which is of direct functional relevance (Wai and Langer, 2016). Mitochondria are highly dynamic, constantly breaking off, spatially relocating, and rejoining the network. This is critical for neurons [particularly dopaminergic neurons, with ~4.5 metres of total linear axonal length and ~2.5 million synapses each (Bolam and Pissadaki, 2012)] as their complex architecture requires mitochondrial relocation from the soma to dendrites, axons and synapses to meet regional metabolic demands (Parrado-Fernandez et al., 2018). The majority of genetic PD proteins locating to mitochondria and mitochondria-ER contact sites are associated with processes influencing or influenced by mitochondrial dynamics and trafficking (Gao et al., 2017; Cutillo et al., 2020). A comprehensive summary of mitochondrial dynamics and trafficking in neuronal function can be found elsewhere (Seager et al., 2020).

Mitochondrial dynamics are highly complex but strictly balanced processes controlled by a diverse array of established and emerging molecular mediators, in addition to mitochondria-ER contact sites (Chan, 2020; Sabouny and Shutt, 2020). Fragmentation of the mitochondrial network is a salient observation in PD, highlighting mitochondrial dynamics as a key therapeutic target. To this end, mdivi-1, an inhibitor of the mitochondrial fission GTPase Drp1, has been used to inhibit mitochondrial fragmentation in an α-synuclein rat model of PD, reducing neurodegeneration, α-synuclein aggregation, mitochondrial dysfunction and oxidative stress (Bido et al., 2017). While therapeutic manipulation of mitochondrial dynamics shows great promise, it will require greater understanding to be effectively exploited.

Mitochondrial motility issues are also common in PD (Smith and Gallo, 2018). Simplistically, mitochondrial trafficking is subject to pernicious cause or consequence cycles, such as (1) reduced energy from mitochondrial dysfunction causing disorganisation of microtubule networks and α-synuclein protein aggregation, or (2) α-synuclein deposition impairing mitochondrial dynamics and axonal trafficking leading to chaotic organellar distribution, synaptic accumulation of autophagosomes and mitochondrial dysfunction (Esteves et al., 2014; Pozo Devoto and Falzone, 2017). The peptide Davenutide has been shown to be neuroprotective in sporadic PD cybrids by stabilising microtubule structure, thus restoring microtubule trafficking, organellar distribution, autophagic flux, mitochondrial membrane potential, as well as reducing α-synuclein accumulation and mitochondrial ubiquitination (Esteves et al., 2014). More recently it has been identified that mitochondria can be transferred between cells, which may be a mechanism for reducing cellular stress, either by enhancing energy production through supplementation of functional mitochondrial to a dysfunctional network or by removing defective organelles from cells with compromised quality control pathways (Shanmughapriya et al., 2020). As such, intercellular mitochondrial transfer may be impaired in PD and could be a worthwhile therapeutic approach. However, this will need careful consideration as it has also been proposed as a potential route by which the neuronal propagation of α-synuclein deposition could occur (Valdinocci et al., 2019).

Pharmacological and genetic targeting of mitochondrial dynamics pathways has been investigated in a number of different disease models (Whitley et al., 2019), yet attempts in PD are limited. Given the complex architecture and minimal metabolic plasticity of neurons, as well as the predominance of mitochondrial dynamics and trafficking defects in genetic and sporadic PD, the preservation, restoration or optimisation of these functions is an important therapeutic consideration for PD and requires further intensive research.

### Addressing Mitochondrial Calcium and Metal Ion Dyshomeostasis

Mitochondrial dysfunction can lead to excitotoxicity through a reduction in cellular ATP levels, an increase in cellular Ca^2+^, or both (Ludtmann and Abramov, 2018). Inhibition of C.I, and consequently ATP generation, lowers intracellular ATP, leading to partial neuronal depolarization due to a reduction in the activity of Na^+^/K^+^-ATPase (Ludtmann and Abramov, 2018). Mitochondria can take up Ca^2+^ from the cytosol via a uniporter transporter, which relies on the mitochondrial membrane potential. The ROS generated by mitochondrial respiratory chain dysfunction can damage the mitochondrial membranes and disrupt this mechanism of Ca^2+^ uptake and storage, thereby raising intracellular Ca^2+^ levels and exacerbating the excitotoxicity (Carbone et al., 2017). Disruption in the mitochondrial membrane potential leads to an increased susceptibility to Ca^2+^ overload. This suggests that mitochondrial-driven excitotoxicity is a major contributory factor in PD (Carbone et al., 2017). Currently, there are no mechanistic treatment approaches available. Based on the ubiquitous role of Ca^2+^ for neuronal signalling, it is unlikely that this pathway will be currently considered as a viable treatment target. However, this might change as we gain a more in-depth insight into the crosslink of mitochondria-related Ca^2+^-dyshomeostasis and the specific vulnerability of dopaminergic neurons.

Mitochondria are responsible for regulating intracellular metal ion [iron (Fe), copper (Cu), and zinc (Zn) levels], their macromolecular organisation (e. g. by forming Fe-Sulphur cluster or heme groups) and their respective distribution in cells (Mezzaroba et al., 2019). Preclinical and human models imply that impaired mitochondrial homeostasis leads to Fe accumulation and Cu deficiency *in vivo* (Tarohda et al., 2005). The SN of PD brains contain increased Fe and decreased Cu levels, which might link the pathology to mitochondrial dysfunction (Tarohda et al., 2005). The increased Fe levels in PD are mainly restricted to the mitochondria (Munoz et al., 2016). A hemiparkinsonian model in monkeys treated with MPTP showed an increase in the nigral iron deposition (Mochizuki et al., 1994), suggesting a functional link between metal ion dyshomeostasis and mitochondrial dysfunction. Also, in these models the Cu level decreased accordingly. One theory assumes that the inhibition of C.I increases the ROS level, which damages the Fe-Sulphur cluster, hinders their proper assembly, and forces the transport of Fe to the mitochondria (Liang and Patel, 2004; Mena et al., 2011). This mechanism results in a vicious circle as the mismatch of divalent and trivalent Fe promotes the formation of oxidative stress. Increased nigral Fe deposition also leads to decreased glutathione levels, which resembles another level of the interconnectedness of mitochondrial dysfunction and Fe-dyshomeostasis (Lee et al., 2009). Chelating agents that can cross the blood-brain barrier (such as deferiprone or deferoxamine) can redistribute Fe ions also on a cellular level (Kakhlon et al., 2010). Deferiprone leads to decreased nigral iron deposition, however, whether decreased Fe deposition also leads to improved mitochondrial function (e. g, by improving mitochondrial bioenergetics) in humans is currently unknown.

The pathophysiological role of Cu in the pathogenesis of PD is only poorly understood. The supplementation of Cu (by Cu-sulphate) seems to be protective against MPTP-induced toxicity in animal models (Rubio-Osornio et al., 2017). Paradoxically, administration of the sequestering Cu-chelator D-penicillamine protects mice from MPTP-related mitochondrial toxicity. In summary, the evaluation of Cu-targeted treatment approaches is limited due to conflicting experimental data and the unknown pathophysiological function.

Zn is likewise disturbed in PD (Park et al., 2015). *ATP13A2*-related parkinsonism may be caused by mitochondrial Zn-aggregation (Park et al., 2014). Besides, mitochondrial toxins have been shown to lead to increased Zn-aggregation in *in-vitro* models (Park and Sue, 2017). Whether treatment regimens targeting Zn provide a fruitful approach for future studies in mitochondrial PD is currently unknown.

In conclusion, preliminary evidence suggests targeting the redistribution of metal ions may be beneficial in PD, with potential neuroprotective effects involving mitochondrial function.

### Targeting the Intersection of Neuroinflammation, Innate Immunity, and Mitochondrial Dysfunction

There is evidence that *LRRK2* mutations contribute to immune alterations both in peripheral organs and the brain, although our understanding of the underlying mechanism is incomplete (Wallings et al., 2020). For example, numerous studies have established that mutations in *LRRK2* confer susceptibility to mycobacterial infection, implying that LRRK2 plays a role in regulating immunity (Zhang et al., 2009; Wang et al., 2015, 2018; Fava et al., 2016).

A recent study demonstrated that loss of LRRK2 in macrophages led to elevated basal levels of type I interferon and interferon stimulated genes, resulting in an attenuated interferon response to mycobacterial pathogens and cytosolic nucleic acid agonists (Weindel et al., 2020). Altered innate immune gene expression in *LRRK2* knockout macrophages is driven by mitochondrial stresses, such as oxidative stress from low levels of purine metabolites and Drp1-dependent mitochondrial fragmentation. This subsequently promotes mtDNA leakage into the cytosol and chronic cyclic GMP-AMP synthase engagement. Although *LRRK2* knockout mice can control Mycobacterium tuberculosis replication, they have exacerbated inflammation and lower interferon stimulated gene expression in the lungs. Thus, while LRRK2 inhibitors are candidate therapies in PD, they may have deleterious outcomes on immune responses. Specifically, they may affect the function of microglia, which are essential for healthy neurons (Weindel et al., 2020).

### Sirtuins

Sirtuins are a family of NAD+ dependent protein deacetylases, with several other enzymatic capabilities (Wang Y. et al., 2019). Sirtuins are associated in general with metabolic regulation and longevity, but are also important in various disease states, including cancer, diabetes, and neurodegeneration (Wang Y. et al., 2019). PD is hallmarked by protein misfolding, mitochondrial dysfunction, oxidative stress and neuroinflammation, all of which are mediated by sirtuins (Zhang et al., 2020). It is therefore reasonable to assume that PD could be modulated by differentially targeting the three predominant sirtuins (SIRT1, 2, and 3), which are of primary relevance to mitochondrial dysfunction in PD (Lin et al., 2018).

In general, SIRT1 and SIRT3 activity is considered neuroprotective in PD, whereas SIRT2 activity appears to be contradictory. In this regard, the focus of therapy has been on enhancing SIRT1 and SIRT3 and inhibiting SIRT2 (Tang, 2017).

Despite the clear involvement of sirtuins in PD, there is a distinct lack of clinical trials investigating their modulation for treatment of neurodegeneration (Bonkowski and Sinclair, 2016; Mautone et al., 2020). This is largely due to a lack of isoform specific inhibitors and activators and the need to target specific cells to avoid unintended effects. Resolution of current controversies and a greater understanding of the tissue-specific functions, as well as the complexity and interconnectedness of sirtuin interaction and activity networks is required to optimally target these molecules (Dang, 2014; Wang Y. et al., 2019; Mautone et al., 2020; Wang et al., 2020).

### miRNAs

Micro RNAs (miRNAs) are non-coding RNA fragments approximately 22 nucleotides in length that modify gene expression. There is considerable complexity in miRNA mediated gene expression given that one gene can be regulated by many miRNAs and one miRNA can influence many genes (Titze-de-Almeida et al., 2020). There is a plethora of literature demonstrating dysregulation of numerous miRNAs in association with PD (Martinez and Peplow, 2017), with consistencies emerging between studies (Goh et al., 2019; Schulz et al., 2019). In fact, most miRNAs associated with PD appear to be linked to mitochondria (Sun et al., 2019; John et al., 2020).

As an example, miRNA181a/b have the capacity to influence mitochondrial biogenesis and quality control, respiratory chain assembly and mitochondrial antioxidants, with downregulation of miRNA181a/b found to be neuroprotective in mitochondrial disorders with neurodegeneration, suggesting a broad gene-independent therapeutic potential for diseases with mitochondrial dysfunction, such as PD (Indrieri et al., 2019).

Nevertheless, a better understanding of the complex positive and negative interplay between different miRNAs and their associated genes is essential. There is therapeutic potential in controlling miRNA expression to modify various mitochondrial pathways, including bioenergetics, biogenesis, oxidative stress, mitophagy, and even cell death (Indrieri et al., 2020). There is also the prospect of developing diagnostic tests, particularly with miRNAs such as miR34b/c, which is altered in the brains of PD patients, even in early pre-motor stages (Minones-Moyano et al., 2011; Cressatti et al., 2020; Ravanidis et al., 2020).

### ER-mitochondrial Links

Mitochondria and the endoplasmic reticulum (ER) are intimately linked by specialised mitochondria-ER contact sites (MERCs), which act as foci of enzymatic activity (Flis and Daum, 2013; Friedman et al., 2018), communication and signalling portals (Takeda and Yanagi, 2019; Reane et al., 2020), and initiation sites (Gelmetti et al., 2017; Elliott et al., 2018). Interactions via MERCs mediate diverse functions, including lipid biosynthesis, mitochondrial dynamics, Ca^2+^ homeostasis, bioenergetics, mitochondrial trafficking, protein folding, ER stress response, autophagy/mitophagy, apoptosis, and inflammation (Gomez-Suaga et al., 2018).

Disruption of MERCs is associated with several neurodegenerative diseases, including PD (Paillusson et al., 2016; Erpapazoglou et al., 2017). A number of proteins associated with mPD (α-synuclein, DJ-1, PINK1, Parkin, LRRK2) are concentrated at MERCs under normal conditions where they mediate diverse, often non-standard, functions (Ottolini et al., 2013; Guardia-Laguarta et al., 2014; Gautier et al., 2016; Gelmetti et al., 2017; Basso et al., 2018; Parrado-Fernandez et al., 2018; Toyofuku et al., 2020), and PD-causing mutations in these proteins are associated with disrupted mitochondria-ER contact and communication.

Although incompletely understood, the contribution of mitochondria-ER dysfunction in PD pathogenesis is evident and targeting MERCs could be of therapeutic benefit. As MERC disruption seems to be ubiquitous across neurodegenerative diseases, any therapies may come from or have utility in several diseases.

### NIX

A putative protective mechanism against PD was identified in an asymptomatic compound heterozygous *PRKN* mutation carrier (Koentjoro et al., 2012). The compound heterozygous (c.8_171del/c.535_871del, p.V3EfsX3/p.G179LfsX7) mutation carrier had not developed PD by her seventh decade despite a complete loss of functional Parkin (Koentjoro et al., 2012). Cells from the asymptomatic carrier showed intact mitochondrial function and mitophagy-mediated by mitochondrial receptor Nip3-like protein X (Nix) (Koentjoro et al., 2017). Furthermore, PINK1 knockdown did not affect Nix-mediated mitophagy. Genetic and pharmacological induction of Nix was able to reestablish mitophagy in *PINK1*- and *PRKN*-related PD patient cell lines, and *Nix* over-expression resulted in an improvement in mitochondrial ATP production. Therefore, Nix could be an alternative mediator of mitophagy and could serve as a neuroprotective therapy in PD due to *PINK1* or *PRKN* mutations (Koentjoro et al., 2017; Naeem et al., 2020).

#### Mitochondrial Transplantation in PD

Improving mitochondrial function by supplementing exogenous mitochondria is a promising strategy in PD (Shanmughapriya et al., 2020). A recent study compared the functionality of mitochondrial transfer with or without Pep-1 conjugation in 6-OHDA-induced PC12 cells and PD rat models (Chang et al., 2016). The investigators injected mitochondria into the medial forebrain bundle of PD rats following a unilateral 6-OHDA lesion. They showed that only peptide-mediated allogeneic mitochondrial delivery with allogeneic and xenogeneic sources preserved mitochondrial function against neurotoxin-induced oxidative stress and apoptotic death in the rat PC12 cells. Additionally, allogeneic and xenogeneic transplantation of peptide-labelled mitochondria improved the locomotive activity in the PD rats (Chang et al., 2016). Another study used a MPTP-induced PD rat model to demonstrate that mitochondria injected intravenously can prevent progression of PD by increasing the activity of the electron transport chain, reducing reactive oxygen species level, and restricting cell apoptosis and necrosis (Shi et al., 2017). These studies support the therapeutic strategy of mitochondrial supplementation injected either directly into the brain or intravenously. The success of mitochondrial transplantation is likely dependent upon the source and quality of the isolated mitochondria, the delivery protocol, as well as the cellular uptake of supplemental mitochondria to ensure adequate neuronal uptake within the brain (Chang et al., 2019).

#### Stem Cell Therapies in PD Addressing Mitochondrial Dysfunction

Astrocytes derived from human iPSCs may be therapeutic in PD via the provision of a continuous supply of healthy mitochondria as a form of mitochondrial donation (Cheng et al., 2020). In favour of this concept, a recent study used a rotenone-induced *in vitro* PD model to demonstrate that iPSCs-derived astrocytes or astrocytic condition media can rescue dopaminergic neurons through intercellular mitochondrial transfer (Cheng et al., 2020).

#### Soluble Epoxide Hydrolase Inhibition

There are multiple lines of evidence suggesting that soluble epoxide hydrolase (sEH) deficiency or inhibition can attenuate parkinsonism in MPTP-treated mice (Qin et al., 2015; Pallas et al., 2020). For example, deficiency and inhibition of sEH attenuates the loss of TH-positive cells and improves rotarod performance (Qin et al., 2015). The substrate of sEH, 14,15-epoxyeicosatrienoic acid (14,15-EET), protected TH-positive cells and alleviated the rotarod performance deficits of wild-type mice, but not sEH-knockout mice. Furthermore, the 14,15-EET antagonist [14,15-epoxyeicosa-5(Z)-enoic acid] abolished the neuronal protective effects of sEH deficiency. In primary cultured cortical neurons, MPP^+^ induces Akt inactivation in neurons from sEH wild-type mice, but not in neurons from knockout mice. This indicates that sEH deficiency and inhibition can increase 14,15-EET in MPTP-treated mice, thereby activating the Akt-mediated protection of TH-positive neurons and behavioural functioning. Thus, sEH inhibition might be a powerful tool to protect dopaminergic neurons in PD (Qin et al., 2015). In addition to having anti-inflammatory properties, EETs may have numerous benefits such as antioxidant effects, a reduction in mitochondrial dysfunction and apoptosis and improved cerebral blood flow (Pallas et al., 2020).

### Photobiomodulation

Photobiomodulation (PBM) refers to the use of light in the red to infrared wavelength range to enhance mitochondrial function by displacing nitric oxide, which competitively binds the molecular oxygen site on C.IV of the respiratory chain (Hamblin, 2018; Quirk and Whelan, 2020). Nevertheless, when applied to stressed cortical neurons with reduced mitochondrial membrane potential, PBM returned the membrane potential to normal, thereby reducing ROS production and excitotoxic Ca^2+^ levels (Huang et al., 2013, 2014), where the effects of PBM on mitochondrial function have been found to be both dose and time dependent (Silveira et al., 2019). In addition to enhancing neurometabolism, PBM has also been found to initiate anti-inflammatory, antioxidant, and anti-apoptotic pathways (Salehpour et al., 2018).

To date over 30 *in vitro* and *in vivo* studies of PBM in PD models have explored variations on the theme (Hong, 2019; Salehpour and Hamblin, 2020). The most comprehensive and compelling research on the neuroprotective effect of PBM in PD has come from studies of transcranial and intracranial PBM in chemically-induced rodents (Peoples et al., 2012; Shaw et al., 2012; Moro et al., 2013, 2014; Purushothuman et al., 2013; O'Callaghan et al., 2015; Reinhart et al., 2015, 2016a,b; Reinhart et al., 2017) and non-human primate PD models (Moro et al., 2016, 2017; El Massri et al., 2017). Evidence suggests that PBM need not be directly applied to neurons, instead showing that non-invasive remote PBM treatment (i.e., to extremities or the abdomen) has a so-called “abscopal” neuroprotective effect (Johnstone et al., 2014; Kim et al., 2018), although the precise mechanisms are still uncertain.

One human pilot trial and one clinical trial of transcranial and intranasal PBM have been conducted to date, which have shown improvements in speech, cognition, gait, and freezing episodes in patients with established PD (Hamilton et al., 2019; Santos et al., 2019). However, the utility of PBM neuroprotection relies on there being neurons to protect in the first instance (Yang et al., 2020), meaning this approach would likely be useful in the early stages of PD with sustained use to moderate disease progression (Foo et al., 2020).

## Discussion

The current clinical diagnosis of Parkinson's disease (PD) is primarily based on the appearance of motor symptoms (Postuma et al., 2015). However, phenotypic changes often manifest several years earlier, driven by early pathogenic processes in a so-called “prodromal” phase (Berg et al., 2015). Symptoms presenting in the prodromal stage are becoming more widely appreciated and highlight a period that may offer a unique window of opportunity for neuroprotective treatment regimens to be administered to avoid or mitigate severe pathology (Postuma and Berg, 2019). However, the underlying aetiology and pathophysiology of PD is complex, hindering the translation of research insights into improved clinical outcomes (Grunewald et al., 2019).

**Figure 3:**
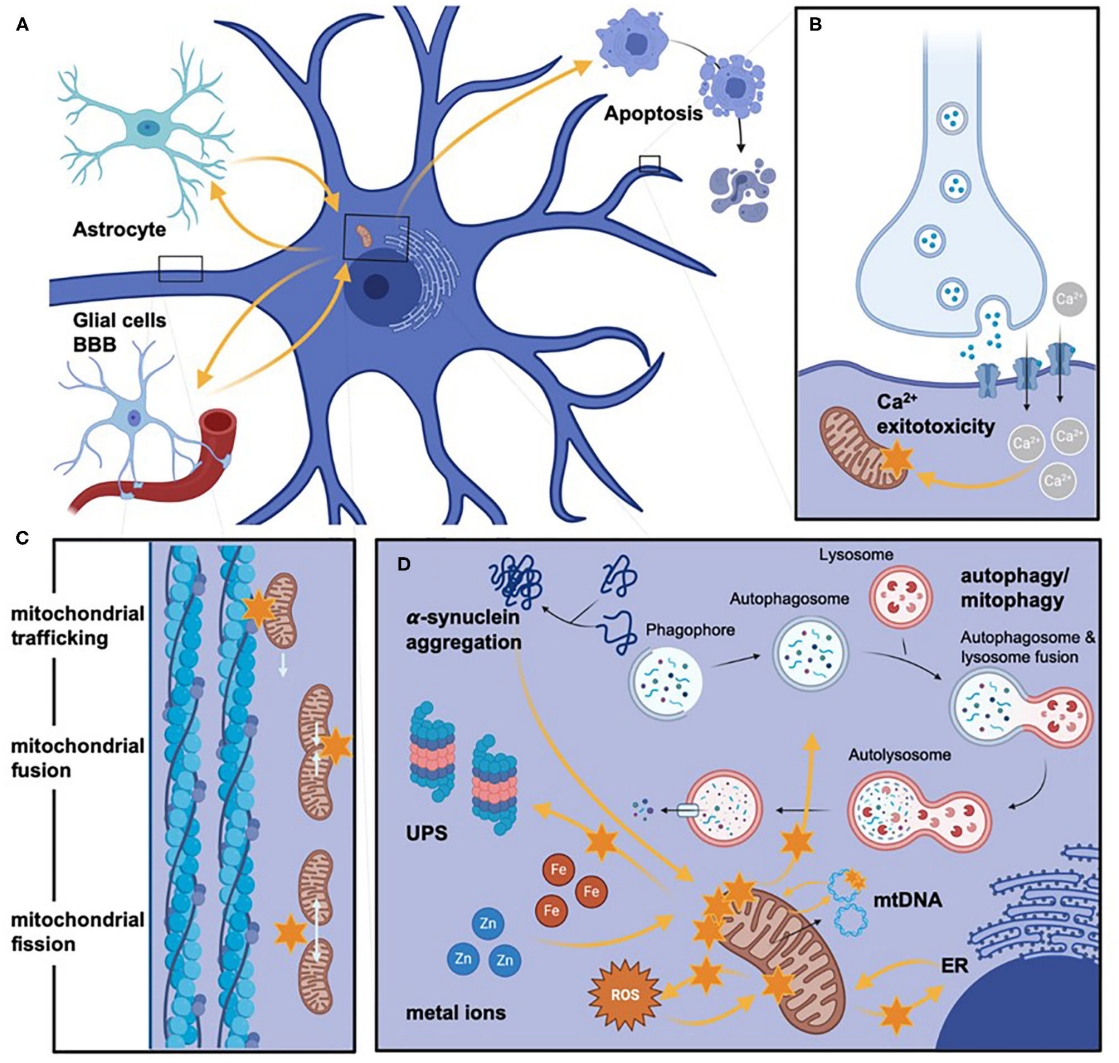
Mitochondrial pathways involved in PD pathogenesis. Different impaired pathways in mitochondrial dysfunction are schematically highlighted but not exhaustively listed. **(A)** Provides an overview of neurons' histopathological orientation and otherwise active cell types (left upper corner: glial cells, left lower corner: pericytes, which form -mong others- the blood-brain barrier, right upper corner: schematic representation of neuronal cell death via apoptosis). Orange arrows are indicating the direct and indirect influence on various factors on mitochondrial dysfunction, which are additionally highlighted by orange stars to stress severe impairment (e.g., the bioenergetic relevance of glial cells for neurons). **(B–D)** Representing more in-depth insights on molecular aspects of mitochondrial dysfunction. **(B)** Illustrates the increased Ca^2+^ influx, which leads to mitochondrial impairment mediated by glutamate-excitotoxicity. **(C)** Displays (represented by a magnified axonal section) alterations of mitochondrial dynamics by impaired mitochondrial trafficking, fusion, or fission. **(D)** Depicts schematically (especially concerning the size of involved cellular components) the commonly shared pathomechanisms of mitochondrial dysfunction. Again, we highlighted the direct and indirect influence on the mitochondria with orange arrows. Even though not all pathomechanisms are named here, the lysosome/autophagosome pathway's involvement and the ubiquitin-proteasome clearance of dysfunctional mitochondria are stated. The influence of toxic cellular compounds is stressed by protein aggregation, Zn and Fe dyshomeostasis, and ROS. In addition, alterations of mtDNA are highlighted. Even though many altered pathways are interconnectedly leading to mitochondrial dysfunction, we only listed the respective influence on mitochondria and, for clarity, not on each other. BBB, blood brain barrier; Ca^2+^, calcium; ER, endoplasmic reticulum; Fe, iron; mtDNA, mitochondrial DNA; ROS, reactive oxygen species; UPS, ubiquitin-proteasome system; Zn, zinc. *Created with* *https://biorender.com/*.

Mitochondrial dysfunction plays a central, multifaceted role in the pathogenesis of PD (Figure 3), whether cause or consequence. It therefore represents an ideal target for candidate drug therapies as its modulation will undoubtedly have an impact on disease progression. However, to date, there has been limited success for clinical trials targeting mitochondrial pathways in PD (Supplementary Table 2). This may be due to a failure to address the multiple interconnected systems and pathways in the highly sensitive homeostatic cellular systems of neurons. Furthermore, it may be difficult to ensure adequate delivery of the drugs to their site of action. Moreover, disease progression in these trials may be too advanced to regain sufficient mitochondrial and therefore target cellular function.

The future of neuroprotective trials will likely rely on combinations of drugs acting directly and indirectly on affected pathways using personalised precision medicine (Titova and Chaudhuri, 2017). Furthermore, it will be necessary to enrich study cohorts for participants that display remarkable dysfunction in these respective pathways and who might benefit the most from targeted treatment approaches (Redensek et al., 2017). It would be helpful to identify these participants early in the prodromal phase to be able to treat prior to advanced stages with neuronal loss. Within this scope, targeting the right patients, with the right treatments, at the right time is the optimal paradigm for clinical PD research.

## Author Contributions

All authors listed have made a substantial, direct and intellectual contribution to the work, and approved it for publication.

## Conflict of Interest

The authors declare that the research was conducted in the absence of any commercial or financial relationships that could be construed as a potential conflict of interest.

## References

[B1] Abdelkader N. F. Safar M. M. Salem H. A. (2016). Ursodeoxycholic acid ameliorates apoptotic cascade in the rotenone model of parkinson's disease: modulation of mitochondrial perturbations. Mol. Neurobiol. 53, 810–817. 10.1007/s12035-014-9043-8 25502462

[B2] Aman Y. Ryan B. Torsetnes S. B. Knapskog A. B. Watne L. O. Mcewan W. A.. (2020). Enhancing mitophagy as a therapeutic approach for neurodegenerative diseases. Int. Rev. Neurobiol. 155, 169–202. 10.1016/bs.irn.2020.02.008 32854854

[B3] Angelova P. R. Abramov A. Y. (2018). Role of mitochondrial ROS in the brain: from physiology to neurodegeneration. FEBS Lett. 592, 692–702. 10.1002/1873-3468.12964 29292494

[B4] Anis E. Zafeer M. F. Firdaus F. Islam S. N. Anees Khan A. Ali A.. (2020). Ferulic acid reinstates mitochondrial dynamics through PGC1alpha expression modulation in 6-hydroxydopamine lesioned rats. Phytother. Res. 34, 214–226. 10.1002/ptr.6523 31657074

[B5] Ascherio A. Schwarzschild M. A. (2016). The epidemiology of Parkinson's disease: risk factors and prevention. Lancet Neurol. 15, 1257–1272. 10.1016/S1474-4422(16)30230-7 27751556

[B6] Athauda D. Maclagan K. Skene S. S. Bajwa-Joseph M. Letchford D. Chowdhury K.. (2017). Exenatide once weekly versus placebo in Parkinson's disease: a randomised, double-blind, placebo-controlled trial. Lancet 390, 1664–1675. 10.1016/S0140-6736(17)31585-4 28781108 PMC5831666

[B7] Attia H. N. Maklad Y. A. (2018). Neuroprotective effects of coenzyme Q10 on paraquat-induced Parkinson's disease in experimental animals. Behav. Pharmacol. 29, 79–86. 10.1097/FBP.0000000000000342 28902670

[B8] Bajracharya R. Ballard J. W. O. (2018). Dietary management and physical exercise can improve climbing defects and mitochondrial activity in drosophila melanogaster parkin null mutants. Fly 12, 95–104. 10.1080/19336934.2018.1482139 30068249 PMC6150633

[B9] Basso V. Marchesan E. Peggion C. Chakraborty J. Von Stockum S. Giacomello M.. (2018). Regulation of ER-mitochondria contacts by Parkin via Mfn2. Pharmacol. Res. 138, 43–56. 10.1016/j.phrs.2018.09.006 30219582

[B10] Bell S. M. Barnes K. Clemmens H. Al-Rafiah A. R. Al-Ofi E. A. Leech V. (2018). Ursodeoxycholic acid improves mitochondrial function and redistributes Drp1 in fibroblasts from patients with either sporadic or familial alzheimer's disease. J. Mol. Biol. 430, 3942–3953. 10.1016/j.jmb.2018.08.019 30171839 PMC6193139

[B11] Bellou V. Belbasis L. Tzoulaki I. Evangelou E. Ioannidis J. P. (2016). Environmental risk factors and Parkinson's disease: an umbrella review of meta-analyses. Parkinsonism Relat. Disord. 23, 1–9. 10.1016/j.parkreldis.2015.12.008 26739246

[B12] Bennett J. P. Jr. Keeney P. M. (2020). Alzheimer's and Parkinson's brain tissues have reduced expression of genes for mtDNA OXPHOS Proteins, mitobiogenesis regulator PGC-1alpha protein and mtRNA stabilizing protein LRPPRC (LRP130). Mitochondrion 53, 154–157. 10.1016/j.mito.2020.05.012 32497722

[B13] Berg D. Postuma R. B. Adler C. H. Bloem B. R. Chan P. Dubois B. (2015). MDS research criteria for prodromal Parkinson's disease. Mov. Disord. 30, 1600–1611. 10.1002/mds.26431 26474317

[B14] Bian M. Liu J. Hong X. Yu M. Huang Y. Sheng Z.. (2012). Overexpression of parkin ameliorates dopaminergic neurodegeneration induced by 1- methyl-4-phenyl-1,2,3,6-tetrahydropyridine in mice. PLoS ONE 7:e39953. 10.1371/journal.pone.0039953 22792139 PMC3390003

[B15] Bido S. Soria F. N. Fan R. Z. Bezard E. Tieu K. (2017). Mitochondrial division inhibitor-1 is neuroprotective in the A53T-alpha-synuclein rat model of Parkinson's disease. Sci. Rep. 7:7495. 10.1038/s41598-017-07181-0 28790323 PMC5548731

[B16] Blauwendraat C. Nalls M. A. Singleton A. B. (2020). The genetic architecture of Parkinson's disease. Lancet Neurol. 19, 170–178. 10.1016/S1474-4422(19)30287-X 31521533 PMC8972299

[B17] Bolam J. P. Pissadaki E. K. (2012). Living on the edge with too many mouths to feed: why dopamine neurons die. Mov. Disord. 27, 1478–1483. 10.1002/mds.25135 23008164 PMC3504389

[B18] Bonkowski M. S. Sinclair D. A. (2016). Slowing ageing by design: the rise of NAD(+) and sirtuin-activating compounds. Nat. Rev. Mol. Cell Biol. 17, 679–690. 10.1038/nrm.2016.93 27552971 PMC5107309

[B19] Borghammer P. Vafaee M. Ostergaard K. Rodell A. Bailey C. Cumming P. (2008). Effect of memantine on CBF and CMRO2 in patients with early Parkinson's disease. Acta Neurol. Scand. 117, 317–323. 10.1111/j.1600-0404.2007.00943.x 17927800

[B20] Bose A. Beal M. F. (2016). Mitochondrial dysfunction in Parkinson's disease. J Neurochem. 139(Suppl 1), 216–231. 10.1111/jnc.13731 27546335

[B21] Bruggemann N. Klein C. (1993). Parkin type of early-onset Parkinson disease, in GeneReviews((R)), eds Adam M. P. Ardinger H. H. Pagon R. A. Wallace S. E. Bean L. J. H. Stephens K. Amemiya A. (Seattle, WA: University of Washington).

[B22] Carbone C. Costa A. Provensi G. Mannaioni G. Masi A. (2017). The Hyperpolarization-activated current determines synaptic excitability, calcium activity and specific viability of substantia nigra dopaminergic neurons. Front. Cell. Neurosci. 11:187. 10.3389/fncel.2017.00187 28701928 PMC5487410

[B23] Chan D. C. (2020). Mitochondrial dynamics and its involvement in disease. Annu. Rev. Pathol. 15, 235–259. 10.1146/annurev-pathmechdis-012419-032711 31585519

[B24] Chandra G. Kundu M. Rangasamy S. B. Dasarathy S. Ghosh S. Watson R.. (2018). Increase in mitochondrial biogenesis in neuronal cells by RNS60, a physically-modified saline, via phosphatidylinositol 3-kinase-mediated upregulation of PGC1alpha. J. Neuroimmune Pharmacol. 13, 143–162. 10.1007/s11481-017-9771-4 29188424 PMC5928179

[B25] Chandra G. Shenoi R. A. Anand R. Rajamma U. Mohanakumar K. P. (2019). Reinforcing mitochondrial functions in aging brain: an insight into Parkinson's disease therapeutics. J. Chem. Neuroanat. 95, 29–42. 10.1016/j.jchemneu.2017.12.004 29269015

[B26] Chang C. Y. Liang M. Z. Chen L. (2019). Current progress of mitochondrial transplantation that promotes neuronal regeneration. Transl. Neurodegener. 8:17. 10.1186/s40035-019-0158-8 31210929 PMC6567446

[B27] Chang J. C. Wu S. L. Liu K. H. Chen Y. H. Chuang C. S. Cheng F. C.. (2016). Allogeneic/xenogeneic transplantation of peptide-labeled mitochondria in Parkinson's disease: restoration of mitochondria functions and attenuation of 6-hydroxydopamine-induced neurotoxicity. Transl. Res. 170, 40.e3–56.e3. 10.1016/j.trsl.2015.12.003 26730494

[B28] Cheng L. Chen L. Wei X. Wang Y. Ren Z. Zeng S.. (2018). NOD2 promotes dopaminergic degeneration regulated by NADPH oxidase 2 in 6-hydroxydopamine model of Parkinson's disease. J. Neuroinflamm. 15:243. 10.1186/s12974-018-1289-z 30157869 PMC6116377

[B29] Cheng X. Y. Biswas S. Li J. Mao C. J. Chechneva O. Chen J.. (2020). Human iPSCs derived astrocytes rescue rotenone-induced mitochondrial dysfunction and dopaminergic neurodegeneration *in vitro* by donating functional mitochondria. Transl. Neurodegener. 9:13. 10.1186/s40035-020-00190-6 32345341 PMC7325238

[B30] Choong C. J. Mochizuki H. (2017). Gene therapy targeting mitochondrial pathway in Parkinson's disease. J. Neural. Transm. 124, 193–207. 10.1007/s00702-016-1616-4 27638713

[B31] Chung E. Choi Y. Park J. Nah W. Park J. Jung Y.. (2020). Intracellular delivery of Parkin rescues neurons from accumulation of damaged mitochondria and pathological alpha-synuclein. Sci Adv. 6:eaba1193. 10.1126/sciadv.aba1193 32494688 PMC7190327

[B32] Clark I. E. Dodson M. W. Jiang C. Cao J. H. Huh J. R. Seol J. H.. (2006). Drosophila pink1 is required for mitochondrial function and interacts genetically with parkin. Nature 441, 1162–1166. 10.1038/nature04779 16672981

[B33] Collaborators G. B. D. N. (2019). Global, regional, and national burden of neurological disorders, 1990-2016: a systematic analysis for the global burden of disease study 2016. Lancet Neurol. 18, 459–480. 10.1016/S1474-4422(18)30499-X 30879893 PMC6459001

[B34] Cressatti M. Juwara L. Galindez J. M. Velly A. M. Nkurunziza E. S. Marier S. (2020). Salivary microR-153 and microR-223 levels as potential diagnostic biomarkers of idiopathic parkinson's disease. Mov. Disord. 35, 468–477. 10.1002/mds.27935 31800144

[B35] Cutillo G. Simon D. K. Eleuteri S. (2020). VPS35 and the mitochondria: connecting the dots in Parkinson's disease pathophysiology. Neurobiol. Dis. 145:105056. 10.1016/j.nbd.2020.105056 32853677

[B36] Dang W. (2014). The controversial world of sirtuins. Drug Discov. Today Technol. 12, e9–e17. 10.1016/j.ddtec.2012.08.003 25027380 PMC4101544

[B37] Davis R. L. Wong S. L. Carling P. J. Payne T. Sue C. M. Bandmann O. (2020). Serum FGF-21, GDF-15, and blood mtDNA copy number are not biomarkers of Parkinson disease. Neurol. Clin. Pract. 10, 40–46. 10.1212/CPJ.0000000000000702 32190419 PMC7057070

[B38] Dolgacheva L. P. Berezhnov A. V. Fedotova E. I. Zinchenko V. P. Abramov A. Y. (2019). Role of DJ-1 in the mechanism of pathogenesis of Parkinson's disease. J. Bioenerg. Biomembr. 51, 175–188. 10.1007/s10863-019-09798-4 31054074 PMC6531411

[B39] Dossi G. Squarcina L. Rango M. (2019). *In vivo* mitochondrial function in idiopathic and genetic parkinson's disease. Metabolites 10:19. 10.3390/metabo10010019 31905632 PMC7023121

[B40] El Massri N. Lemgruber A. P. Rowe I. J. Moro C. Torres N. Reinhart F.. (2017). Photobiomodulation-induced changes in a monkey model of Parkinson's disease: changes in tyrosine hydroxylase cells and GDNF expression in the striatum. Exp. Brain Res. 235, 1861–1874. 10.1007/s00221-017-4937-0 28299414

[B41] Elliott E. I. Miller A. N. Banoth B. Iyer S. S. Stotland A. Weiss J. P.. (2018). Cutting edge: mitochondrial assembly of the NLRP3 inflammasome complex is initiated at priming. J. Immunol. 200, 3047–3052. 10.4049/jimmunol.1701723 29602772 PMC5916517

[B42] Erpapazoglou Z. Mouton-Liger F. Corti O. (2017). From dysfunctional endoplasmic reticulum-mitochondria coupling to neurodegeneration. Neurochem. Int. 109, 171–183. 10.1016/j.neuint.2017.03.021 28389271

[B43] Esteves A. R. Gozes I. Cardoso S. M. (2014). The rescue of microtubule-dependent traffic recovers mitochondrial function in Parkinson's disease. Biochim. Biophys. Acta 1842, 7–21. 10.1016/j.bbadis.2013.10.003 24120997

[B44] Fan R. Li X. Gu X. Chan J. C. Xu G. (2010). Exendin-4 protects pancreatic beta cells from human islet amyloid polypeptide-induced cell damage: potential involvement of AKT and mitochondria biogenesis. Diabetes Obes. Metab. 12, 815–824. 10.1111/j.1463-1326.2010.01238.x 20649634

[B45] Fava V. M. Manry J. Cobat A. Orlova M. Van Thuc N. Ba N. N.. (2016). A Missense LRRK2 variant is a risk factor for excessive inflammatory responses in leprosy. PLoS Negl. Trop. Dis. 10:e0004412. 10.1371/journal.pntd.0004412 26844546 PMC4742274

[B46] Fernandes Ferreira A. F. Binda K. H. Singulani M. P. Pereira C. P. M. Ferrari G. D. Alberici L. C.. (2020). Physical exercise protects against mitochondria alterations in the 6-hidroxydopamine rat model of Parkinson's disease. Behav. Brain Res. 387:112607. 10.1016/j.bbr.2020.112607 32199987

[B47] Flis V. V. Daum G. (2013). Lipid transport between the endoplasmic reticulum and mitochondria. Cold Spring Harb. Perspect. Biol. 5:a013235. 10.1101/cshperspect.a013235 23732475 PMC3660828

[B48] Foo A. S. C. Soong T. W. Yeo T. T. Lim K. L. (2020). Mitochondrial dysfunction and parkinson's disease-near-infrared photobiomodulation as a potential therapeutic strategy. Front. Aging Neurosci. 12:89. 10.3389/fnagi.2020.00089 32308618 PMC7145956

[B49] Forester B. P. Berlow Y. A. Harper D. G. Jensen J. E. Lange N. Froimowitz M. P.. (2010). Age-related changes in brain energetics and phospholipid metabolism. NMR Biomed. 23, 242–250. 10.1002/nbm.1444 19908224

[B50] Friedman J. R. Kannan M. Toulmay A. Jan C. H. Weissman J. S. Prinz W. A.. (2018). Lipid homeostasis is maintained by dual targeting of the mitochondrial PE biosynthesis enzyme to the ER. Dev. Cell 44, 261.e6–227.e6. 10.1016/j.devcel.2017.11.023 29290583 PMC5975648

[B51] Gao J. Wang L. Liu J. Xie F. Su B. Wang X. (2017). Abnormalities of mitochondrial dynamics in neurodegenerative diseases. Antioxidants 6:25. 10.3390/antiox6020025 28379197 PMC5488005

[B52] Gautier C. A. Erpapazoglou Z. Mouton-Liger F. Muriel M. P. Cormier F. Bigou S.. (2016). The endoplasmic reticulum-mitochondria interface is perturbed in PARK2 knockout mice and patients with PARK2 mutations. Hum. Mol. Genet. 25, 2972–2984. 10.1093/hmg/ddw148 27206984

[B53] Ge P. Dawson V. L. Dawson T. M. (2020). PINK1 and Parkin mitochondrial quality control: a source of regional vulnerability in Parkinson's disease. Mol. Neurodegener. 15:20. 10.1186/s13024-020-00367-7 32169097 PMC7071653

[B54] Gelmetti V. De Rosa P. Torosantucci L. Marini E. S. Romagnoli A. Di Rienzo M.. (2017). PINK1 and BECN1 relocalize at mitochondria-associated membranes during mitophagy and promote ER-mitochondria tethering and autophagosome formation. Autophagy 13, 654–669. 10.1080/15548627.2016.1277309 28368777 PMC5388214

[B55] Germuska M. Chandler H. L. Stickland R. C. Foster C. Fasano F. Okell T. W.. (2019). Dual-calibrated fMRI measurement of absolute cerebral metabolic rate of oxygen consumption and effective oxygen diffusivity. Neuroimage 184, 717–728. 10.1016/j.neuroimage.2018.09.035 30278214 PMC6264385

[B56] Ghosh A. Langley M. R. Harischandra D. S. Neal M. L. Jin H. Anantharam V.. (2016). Mitoapocynin treatment protects against neuroinflammation and dopaminergic neurodegeneration in a preclinical animal model of parkinson's disease. J. Neuroimmune Pharmacol. 11, 259–278. 10.1007/s11481-016-9650-4 26838361 PMC4995106

[B57] Goh S. Y. Chao Y. X. Dheen S. T. Tan E. K. Tay S. S. (2019). Role of microRNAs in parkinson's disease. Int. J. Mol. Sci. 20:65. 10.3390/ijms20225649 31718095 PMC6888719

[B58] Golpich M. Amini E. Mohamed Z. Azman Ali R. Mohamed Ibrahim N. Ahmadiani A. (2017). Mitochondrial dysfunction and biogenesis in neurodegenerative diseases: pathogenesis and treatment. CNS Neurosci. Ther. 23, 5–22. 10.1111/cns.12655 27873462 PMC6492703

[B59] Gomez-Suaga P. Bravo-San Pedro J. M. Gonzalez-Polo R. A. Fuentes J. M. Niso-Santano M. (2018). ER-mitochondria signaling in Parkinson's disease. Cell Death Dis. 9:337 10.1038/s41419-017-0079-3 29497039 PMC5832754

[B60] Grimm A. Eckert A. (2017). Brain aging and neurodegeneration: from a mitochondrial point of view. J. Neurochem. 143, 418–431. 10.1111/jnc.14037 28397282 PMC5724505

[B61] Grunewald A. Kumar K. R. Sue C. M. (2019). New insights into the complex role of mitochondria in Parkinson's disease. Prog. Neurobiol. 177, 73–93. 10.1016/j.pneurobio.2018.09.003 30219247

[B62] Guardia-Laguarta C. Area-Gomez E. Rub C. Liu Y. Magrane J. Becker D.. (2014). alpha-Synuclein is localized to mitochondria-associated ER membranes. J. Neurosci. 34, 249–259. 10.1523/JNEUROSCI.2507-13.2014 24381286 PMC3866487

[B63] Gureev A. P. Popov V. N. (2019). Nrf2/ARE pathway as a therapeutic target for the treatment of parkinson diseases. Neurochem. Res. 44, 2273–2279. 10.1007/s11064-018-02711-2 30617864

[B64] Hamblin M. R. (2018). Mechanisms and Mitochondrial Redox Signaling in Photobiomodulation. Photochem. Photobiol. 94, 199–212. 10.1111/php.12864 29164625 PMC5844808

[B65] Hamilton C. L. El Khoury H. Hamilton D. Nicklason F. Mitrofanis J. (2019). “Buckets”: early observations on the use of red and infrared light helmets in parkinson's disease patients. Photobiomodul. Photomed. Laser Surg. 37, 615–622. 10.1089/photob.2019.4663 31536464

[B66] Haque M. E. Mount M. P. Safarpour F. Abdel-Messih E. Callaghan S. Mazerolle C. (2012). Inactivation of Pink1 gene *in vivo* sensitizes dopamine-producing neurons to 1-methyl-4-phenyl-1,2,3,6-tetrahydropyridine (MPTP) and can be rescued by autosomal recessive Parkinson disease genes, Parkin or DJ-1. J. Biol. Chem. 287, 23162–23170. 10.1074/jbc.M112.346437 22511790 PMC3391096

[B67] Haque M. E. Thomas K. J. D'souza C. Callaghan S. Kitada T. Slack R. S.. (2008). Cytoplasmic Pink1 activity protects neurons from dopaminergic neurotoxin MPTP. Proc. Natl. Acad. Sci. U.S.A. 105, 1716–1721. 10.1073/pnas.0705363105 18218782 PMC2234210

[B68] He L. Zhou Q. Huang Z. Xu J. Zhou H. Lv D.. (2019). PINK1/Parkin-mediated mitophagy promotes apelin-13-induced vascular smooth muscle cell proliferation by AMPKalpha and exacerbates atherosclerotic lesions. J. Cell. Physiol. 234, 8668–8682. 10.1002/jcp.27527 30456860

[B69] Henchcliffe C. Shungu D. C. Mao X. Huang C. Nirenberg M. J. Jenkins B. G.. (2008). Multinuclear magnetic resonance spectroscopy for *in vivo* assessment of mitochondrial dysfunction in Parkinson's disease. Ann. N. Y. Acad. Sci. 1147, 206–220. 10.1196/annals.1427.037 19076443

[B70] Hong N. (2019). Photobiomodulation as a treatment for neurodegenerative disorders: current and future trends. Biomed. Eng. Lett. 9, 359–366. 10.1007/s13534-019-00115-x 31456895 PMC6694374

[B71] Huang Y. Y. Nagata K. Tedford C. E. Hamblin M. R. (2014). Low-level laser therapy (810 nm) protects primary cortical neurons against excitotoxicity *in vitro*. J. Biophotonics 7, 656–664. 10.1002/jbio.201300125 24127337 PMC4057365

[B72] Huang Y. Y. Nagata K. Tedford C. E. Mccarthy T. Hamblin M. R. (2013). Low-level laser therapy (LLLT) reduces oxidative stress in primary cortical neurons *in vitro*. J. Biophotonics 6, 829–838. 10.1002/jbio.201200157 23281261 PMC3651776

[B73] Indrieri A. Carrella S. Carotenuto P. Banfi S. Franco B. (2020). The pervasive role of the MIR-181 family in development, neurodegeneration, and cancer. Int. J. Mol. Sci. 21:2092. 10.3390/ijms21062092 32197476 PMC7139714

[B74] Indrieri A. Carrella S. Romano A. Spaziano A. Marrocco E. Fernandez-Vizarra E.. (2019). miR-181a/b downregulation exerts a protective action on mitochondrial disease models. EMBO Mol. Med. 11:e8734. 10.15252/emmm.201708734 30979712 PMC6505685

[B75] Inose Y. Izumi Y. Takada-Takatori Y. Akaike A. Koyama Y. Kaneko S.. (2020). Protective effects of Nrf2-ARE activator on dopaminergic neuronal loss in Parkinson disease model mice: possible involvement of heme oxygenase-1. Neurosci. Lett. 736:135268. 10.1016/j.neulet.2020.135268 32712353

[B76] Izumi Y. Sawada H. Yamamoto N. Kume T. Katsuki H. Shimohama S.. (2007). Novel neuroprotective mechanisms of pramipexole, an anti-Parkinson drug, against endogenous dopamine-mediated excitotoxicity. Eur. J. Pharmacol. 557, 132–140. 10.1016/j.ejphar.2006.11.011 17161393

[B77] Jang Y. Kwon I. Song W. Cosio-Lima L. M. Taylor S. Lee Y. (2018). Modulation of mitochondrial phenotypes by endurance exercise contributes to neuroprotection against a MPTP-induced animal model of PD. Life Sci. 209, 455–465. 10.1016/j.lfs.2018.08.045 30144449

[B78] John A. Kubosumi A. Reddy P. H. (2020). Mitochondrial MicroRNAs in aging and neurodegenerative diseases. Cells 9:1345. 10.3390/cells9061345 32481587 PMC7349858

[B79] Johnstone D. M. El Massri N. Moro C. Spana S. Wang X. S. Torres N.. (2014). Indirect application of near infrared light induces neuroprotection in a mouse model of parkinsonism - an abscopal neuroprotective effect. Neuroscience 274, 93–101. 10.1016/j.neuroscience.2014.05.023 24857852

[B80] Kakhlon O. Breuer W. Munnich A. Cabantchik Z. I. (2010). Iron redistribution as a therapeutic strategy for treating diseases of localized iron accumulation. Can. J. Physiol. Pharmacol. 88, 187–196. 10.1139/Y09-128 20393584

[B81] Keeney P. M. Quigley C. K. Dunham L. D. Papageorge C. M. Iyer S. Thomas R. R.. (2009). Mitochondrial gene therapy augments mitochondrial physiology in a Parkinson's disease cell model. Hum. Gene Ther. 20, 897–907. 10.1089/hum.2009.023 19374590 PMC2829286

[B82] Kim B. Mitrofanis J. Stone J. Johnstone D. M. (2018). Remote tissue conditioning is neuroprotective against MPTP insult in mice. IBRO Rep. 4, 14–17. 10.1016/j.ibror.2018.01.001 30135947 PMC6084900

[B83] Koentjoro B. Park J. S. Ha A. D. Sue C. M. (2012). Phenotypic variability of parkin mutations in single kindred. Mov. Disord. 27, 1299–1303. 10.1002/mds.25041 22807239

[B84] Koentjoro B. Park J. S. Sue C. M. (2017). Nix restores mitophagy and mitochondrial function to protect against PINK1/Parkin-related Parkinson's disease. Sci. Rep. 7:44373. 10.1038/srep44373 28281653 PMC5345073

[B85] Krumpolec P. Vallova S. Slobodova L. Tirpakova V. Vajda M. Schon M.. (2017). Aerobic-strength exercise improves metabolism and clinical state in parkinson's disease patients. Front. Neurol. 8:698. 10.3389/fneur.2017.00698 29312123 PMC5743754

[B86] Kumar M. Acevedo-Cintron J. Jhaldiyal A. Wang H. Andrabi S. A. Eacker S.. (2020). Defects in mitochondrial biogenesis drive mitochondrial alterations in PARKIN-deficient human dopamine neurons. Stem Cell Rep. 15, 629–645. 10.1016/j.stemcr.2020.07.013 32795422 PMC7486221

[B87] Kumar S. Rao R. Kumar A. Mahant S. Nanda S. (2016). Novel carriers for coenzyme Q10 delivery. Curr. Drug Deliv. 13, 1184–1204. 10.2174/1567201813666160104130631 26725722

[B88] Lai J. H. Chen K. Y. Wu J. C. Olson L. Brene S. Huang C. Z.. (2019). Voluntary exercise delays progressive deterioration of markers of metabolism and behavior in a mouse model of Parkinson's disease. Brain Res. 1720:146301. 10.1016/j.brainres.2019.146301 31226324 PMC6702069

[B89] Lange F. Dunne L. Hale L. Tachtsidis I. (2019). MAESTROS: A multiwavelength time-domain NIRS system to monitor changes in oxygenation and oxidation state of cytochrome-C-oxidase. IEEE J. Sel. Top. Quantum. Electron. 25:7100312. 10.1109/JSTQE.2018.2833205 30450021 PMC6054019

[B90] Langley M. Ghosh A. Charli A. Sarkar S. Ay M. Luo J.. (2017). Mito-apocynin prevents mitochondrial dysfunction, microglial activation, oxidative damage, and progressive neurodegeneration in mitopark transgenic mice. Antioxid. Redox Signal 27, 1048–1066. 10.1089/ars.2016.6905 28375739 PMC5651937

[B91] Langston J. W. (2017). The MPTP story. J. Parkinsons. Dis. 7, S11–S19. 10.3233/JPD-179006 28282815 PMC5345642

[B92] Lara R. S. Matson G. B. Hugg J. W. Maudsley A. A. Weiner M. W. (1993). Quantitation of *in vivo* phosphorus metabolites in human brain with magnetic resonance spectroscopic imaging (MRSI). Magn. Reson. Imaging 11, 273–278. 10.1016/0730-725X(93)90033-A 8455438

[B93] Larsen S. B. Hanss Z. Kruger R. (2018). The genetic architecture of mitochondrial dysfunction in Parkinson's disease. Cell Tissue Res. 373, 21–37. 10.1007/s00441-017-2768-8 29372317 PMC6015629

[B94] Lee D. W. Kaur D. Chinta S. J. Rajagopalan S. Andersen J. K. (2009). A disruption in iron-sulfur center biogenesis via inhibition of mitochondrial dithiol glutaredoxin 2 may contribute to mitochondrial and cellular iron dysregulation in mammalian glutathione-depleted dopaminergic cells: implications for Parkinson's disease. Antioxid. Redox Signal 11, 2083–2094. 10.1089/ars.2009.2489 19290777 PMC2819798

[B95] Lehmann S. Loh S. H. Martins L. M. (2017). Enhancing NAD^+^ salvage metabolism is neuroprotective in a PINK1 model of Parkinson's disease. Biol. Open 6, 141–147. 10.1242/bio.022186 28011627 PMC5312101

[B96] Liang L. P. Patel M. (2004). Iron-sulfur enzyme mediated mitochondrial superoxide toxicity in experimental Parkinson's disease. J. Neurochem. 90, 1076–1084. 10.1111/j.1471-4159.2004.02567.x 15312163

[B97] Lin M. T. Cantuti-Castelvetri I. Zheng K. Jackson K. E. Tan Y. B. Arzberger T.. (2012). Somatic mitochondrial DNA mutations in early Parkinson and incidental Lewy body disease. Ann. Neurol. 71, 850–854. 10.1002/ana.23568 22718549 PMC3383820

[B98] Lin M. W. Lin C. C. Chen Y. H. Yang H. B. Hung S. Y. (2019). Celastrol inhibits dopaminergic neuronal death of parkinson's disease through activating mitophagy. Antioxidants 9:37. 10.3390/antiox9010037 31906147 PMC7022523

[B99] Lin S. Xing H. Zang T. Ruan X. Wo L. He M. (2018). Sirtuins in mitochondrial stress: Indispensable helpers behind the scenes. Ageing Res. Rev. 44, 22–32. 10.1016/j.arr.2018.03.006 29580919

[B100] Liu S. Lu B. (2010). Reduction of protein translation and activation of autophagy protect against PINK1 pathogenesis in drosophila melanogaster. PLoS Genet. 6:e1001237. 10.1371/journal.pgen.1001237 21151574 PMC3000346

[B101] Liu S. Sawada T. Lee S. Yu W. Silverio G. Alapatt P.. (2012). Parkinson's disease-associated kinase PINK1 regulates miro protein level and axonal transport of mitochondria. PLoS Genet. 8:e1002537. 10.1371/journal.pgen.1002537 22396657 PMC3291531

[B102] Liu X. L. Wang Y. D. Yu X. M. Li D. W. Li G. R. (2018). Mitochondria-mediated damage to dopaminergic neurons in Parkinson's disease (Review). Int. J. Mol. Med. 41, 615–623. 10.3892/ijmm.2017.3255 29207041

[B103] Ludtmann M. H. R. Abramov A. Y. (2018). Mitochondrial calcium imbalance in Parkinson's disease. Neurosci. Lett. 663, 86–90. 10.1016/j.neulet.2017.08.044 28838811

[B104] Ma L. Mao W. Xu E. Cai Y. Wang C. Chhetri J. K.. (2020). Novel POLG mutation in a patient with early-onset parkinsonism, progressive external ophthalmoplegia and optic atrophy. Int. J. Neurosci. 130, 319–321. 10.1080/00207454.2019.1681422 31613174

[B105] Martinez B. Peplow P. V. (2017). MicroRNAs in Parkinson's disease and emerging therapeutic targets. Neural Regen Res. 12, 1945–1959. 10.4103/1673-5374.221147 29323027 PMC5784336

[B106] Mautone N. Zwergel C. Mai A. Rotili D. (2020). Sirtuin modulators: where are we now? a review of patents from 2015 to 2019. Expert Opin. Ther. Pat. 30, 389–407. 10.1080/13543776.2020.1749264 32228181

[B107] Mena N. P. Bulteau A. L. Salazar J. Hirsch E. C. Nunez M. T. (2011). Effect of mitochondrial complex I inhibition on Fe-S cluster protein activity. Biochem. Biophys. Res. Commun. 409, 241–246. 10.1016/j.bbrc.2011.04.137 21570952

[B108] Mezzaroba L. Alfieri D. F. Colado Simao A. N. Vissoci Reiche E. M. (2019). The role of zinc, copper, manganese and iron in neurodegenerative diseases. Neurotoxicology 74, 230–241. 10.1016/j.neuro.2019.07.007 31377220

[B109] Minones-Moyano E. Porta S. Escaramis G. Rabionet R. Iraola S. Kagerbauer B.. (2011). MicroRNA profiling of Parkinson's disease brains identifies early downregulation of miR-34b/c which modulate mitochondrial function. Hum. Mol. Genet. 20, 3067–3078. 10.1093/hmg/ddr210 21558425

[B110] Mischley L. K. Conley K. E. Shankland E. G. Kavanagh T. J. Rosenfeld M. E. Duda J. E.. (2016). Central nervous system uptake of intranasal glutathione in Parkinson's disease. NPJ Parkinsons Dis. 2:16002. 10.1038/npjparkd.2016.2 28725693 PMC5516583

[B111] Mischley L. K. Lau R. C. Shankland E. G. Wilbur T. K. Padowski J. M. (2017). Phase IIb study of intranasal glutathione in parkinson's disease. J. Parkinsons. Dis. 7, 289–299. 10.3233/JPD-161040 28436395 PMC5438472

[B112] Mishra A. Singh S. Tiwari V. Bano S. Shukla S. (2020). Dopamine D1 receptor agonism induces dynamin related protein-1 inhibition to improve mitochondrial biogenesis and dopaminergic neurogenesis in rat model of Parkinson's disease. Behav. Brain Res. 378:112304. 10.1016/j.bbr.2019.112304 31626851

[B113] Mochizuki H. Imai H. Endo K. Yokomizo K. Murata Y. Hattori N.. (1994). Iron accumulation in the substantia nigra of 1-methyl-4-phenyl-1,2,3,6-tetrahydropyridine (MPTP)-induced hemiparkinsonian monkeys. Neurosci. Lett. 168, 251–253. 10.1016/0304-3940(94)90462-6 8028787

[B114] Mok B. Y. De Moraes M. H. Zeng J. Bosch D. E. Kotrys A. V. Raguram A.. (2020). A bacterial cytidine deaminase toxin enables CRISPR-free mitochondrial base editing. Nature 583, 631–637. 10.1038/s41586-020-2477-4 32641830 PMC7381381

[B115] Monti D. A. Zabrecky G. Kremens D. Liang T. W. Wintering N. A. Bazzan A. J.. (2019). N-Acetyl cysteine is associated with dopaminergic improvement in Parkinson's disease. Clin. Pharmacol. Ther. 106, 884–890. 10.1002/cpt.1548 31206613

[B116] Moro C. El Massri N. Darlot F. Torres N. Chabrol C. Agay D.. (2016). Effects of a higher dose of near-infrared light on clinical signs and neuroprotection in a monkey model of Parkinson's disease. Brain Res. 1648, 19–26. 10.1016/j.brainres.2016.07.005 27396907

[B117] Moro C. Massri N. E. Torres N. Ratel D. De Jaeger X. Chabrol C.. (2014). Photobiomodulation inside the brain: a novel method of applying near-infrared light intracranially and its impact on dopaminergic cell survival in MPTP-treated mice. J. Neurosurg. 120, 670–683. 10.3171/2013.9.JNS13423 24160475

[B118] Moro C. Torres N. Arvanitakis K. Cullen K. Chabrol C. Agay D.. (2017). No evidence for toxicity after long-term photobiomodulation in normal non-human primates. Exp. Brain Res. 235, 3081–3092. 10.1007/s00221-017-5048-7 28744621

[B119] Moro C. Torres N. El Massri N. Ratel D. Johnstone D. M. Stone J.. (2013). Photobiomodulation preserves behaviour and midbrain dopaminergic cells from MPTP toxicity: evidence from two mouse strains. BMC Neurosci. 14:40. 10.1186/1471-2202-14-40 23531041 PMC3616839

[B120] Mortiboys H. Furmston R. Bronstad G. Aasly J. Elliott C. Bandmann O. (2015). UDCA exerts beneficial effect on mitochondrial dysfunction in LRRK2(G2019S) carriers and *in vivo*. Neurology 85, 846–852. 10.1212/WNL.0000000000001905 26253449 PMC4560055

[B121] Munoz Y. Carrasco C. M. Campos J. D. Aguirre P. Nunez M. T. (2016). Parkinson's Disease: the mitochondria-iron link. Parkinsons. Dis. 2016:7049108. 10.1155/2016/7049108 27293957 PMC4886095

[B122] Murphy M. P. Hartley R. C. (2018). Mitochondria as a therapeutic target for common pathologies. Nat. Rev. Drug Discov. 17, 865–886. 10.1038/nrd.2018.174 30393373

[B123] Naeem S. Qi Y. Tian Y. Zhang Y. (2020). NIX compensates lost role of parkin in cd-induced mitophagy in HeLa cells through phosphorylation. Toxicol. Lett. 326, 1–10. 10.1016/j.toxlet.2020.03.001 32142837

[B124] Negida A. Menshawy A. El Ashal G. Elfouly Y. Hani Y. Hegazy Y.. (2016). Coenzyme Q10 for patients with parkinson's disease: a systematic review and meta-analysis. CNS Neurol. Disord. Drug Targets 15, 45–53. 10.2174/1871527314666150821103306 26553164

[B125] O'Callaghan M. M. Emperador S. Pineda M. Lopez-Gallardo E. Montero R. Yubero D.. (2015). Mutation loads in different tissues from six pathogenic mtDNA point mutations. Mitochondrion 22, 17–22. 10.1016/j.mito.2015.03.001 25765153

[B126] O'Connor D. M. Boulis N. M. (2015). Gene therapy for neurodegenerative diseases. Trends Mol. Med. 21, 504–512. 10.1016/j.molmed.2015.06.001 26122838

[B127] Orr A. L. Rutaganira F. U. De Roulet D. Huang E. J. Hertz N. T. Shokat K. M.. (2017). Long-term oral kinetin does not protect against alpha-synuclein-induced neurodegeneration in rodent models of Parkinson's disease. Neurochem. Int. 109, 106–116. 10.1016/j.neuint.2017.04.006 28434973 PMC5641232

[B128] Ottolini D. Cali T. Negro A. Brini M. (2013). The Parkinson disease-related protein DJ-1 counteracts mitochondrial impairment induced by the tumour suppressor protein p53 by enhancing endoplasmic reticulum-mitochondria tethering. Hum. Mol. Genet. 22, 2152–2168. 10.1093/hmg/ddt068 23418303

[B129] Paillusson S. Stoica R. Gomez-Suaga P. Lau D. H. W. Mueller S. Miller T.. (2016). There's something wrong with my MAM; the ER-mitochondria axis and neurodegenerative diseases. Trends Neurosci. 39, 146–157. 10.1016/j.tins.2016.01.008 26899735 PMC4780428

[B130] Pallas M. Vazquez S. Sanfeliu C. Galdeano C. Grinan-Ferre C. (2020). Soluble epoxide hydrolase inhibition to face neuroinflammation in parkinson's disease: a new therapeutic strategy. Biomolecules 10:703. 10.3390/biom10050703 32369955 PMC7277900

[B131] Park J. S. Blair N. F. Sue C. M. (2015). The role of ATP13A2 in Parkinson's disease: clinical phenotypes and molecular mechanisms. Mov. Disord. 30, 770–779. 10.1002/mds.26243 25900096

[B132] Park J. S. Davis R. L. Sue C. M. (2018). Mitochondrial dysfunction in parkinson's disease: new mechanistic insights and therapeutic perspectives. Curr. Neurol. Neurosci. Rep. 18:21. 10.1007/s11910-018-0829-3 29616350 PMC5882770

[B133] Park J. S. Koentjoro B. Davis R. L. Sue C. M. (2016). Loss of ATP13A2 impairs glycolytic function in Kufor-Rakeb syndrome patient-derived cell models. Parkinsonism Relat. Disord. 27, 67–73. 10.1016/j.parkreldis.2016.03.018 27039055

[B134] Park J. S. Koentjoro B. Veivers D. Mackay-Sim A. Sue C. M. (2014). Parkinson's disease-associated human ATP13A2 (PARK9) deficiency causes zinc dyshomeostasis and mitochondrial dysfunction. Hum. Mol. Genet. 23, 2802–2815. 10.1093/hmg/ddt623 24399444 PMC4014187

[B135] Park J. S. Sue C. M. (2017). Hereditary parkinsonism-associated genetic variations in PARK9 locus lead to functional impairment of ATPase Type 13A2. Curr. Protein Pept. Sci. 18, 725–732. 10.2174/1389203717666160311121534 26965689

[B136] Parrado-Fernandez C. Schneider B. Ankarcrona M. Conti M. M. Cookson M. R. Kivipelto M.. (2018). Reduction of PINK1 or DJ-1 impair mitochondrial motility in neurites and alter ER-mitochondria contacts. J. Cell. Mol. Med. 22, 5439–5449. 10.1111/jcmm.13815 30133157 PMC6201361

[B137] Peoples C. Spana S. Ashkan K. Benabid A. L. Stone J. Baker G. E.. (2012). Photobiomodulation enhances nigral dopaminergic cell survival in a chronic MPTP mouse model of Parkinson's disease. Parkinsonism Relat. Disord. 18, 469–476. 10.1016/j.parkreldis.2012.01.005 22285756

[B138] Pickrell A. M. Youle R. J. (2015). The roles of PINK1, parkin, and mitochondrial fidelity in Parkinson's disease. Neuron 85, 257–273. 10.1016/j.neuron.2014.12.007 25611507 PMC4764997

[B139] Postuma R. B. Berg D. (2019). Prodromal Parkinson's disease: the decade past, the decade to come. Mov. Disord. 34, 665–675. 10.1002/mds.27670 30919499

[B140] Postuma R. B. Berg D. Stern M. Poewe W. Olanow C. W. Oertel W. (2015). MDS clinical diagnostic criteria for Parkinson's disease. Mov. Disord. 30, 1591–1601. 10.1002/mds.26424 26474316

[B141] Pozo Devoto V. M. Falzone T. L. (2017). Mitochondrial dynamics in Parkinson's disease: a role for alpha-synuclein? Dis. Model. Mech. 10, 1075–1087. 10.1242/dmm.026294 28883016 PMC5611962

[B142] Prasuhn J. Brüggemann N. Hessler N. Berg D. Gasser T. Brockmann K.. (2019). An omics-based strategy using coenzyme Q10 in patients with Parkinson's disease: concept evaluation in a double-blind randomized placebo-controlled parallel group trial. Neurol. Res. Prac. 1:31. 10.1186/s42466-019-0033-1 33324897 PMC7650116

[B143] Purushothuman S. Nandasena C. Johnstone D. M. Stone J. Mitrofanis J. (2013). The impact of near-infrared light on dopaminergic cell survival in a transgenic mouse model of parkinsonism. Brain Res. 1535, 61–70. 10.1016/j.brainres.2013.08.047 23998985

[B144] Qin X. Wu Q. Lin L. Sun A. Liu S. Li X. (2015). Soluble epoxide hydrolase deficiency or inhibition attenuates MPTP-induced parkinsonism. Mol. Neurobiol. 52, 187–195. 10.1007/s12035-014-8833-3 25128026

[B145] Quirk B. J. Whelan H. T. (2020). What lies at the heart of photobiomodulation: light, cytochrome c oxidase, and nitric oxide-review of the evidence. Photobiomodul. Photomed. Laser Surg. 38, 527–530. 10.1089/photob.2020.4905 32716711 PMC7495914

[B146] Ramalingam M. Huh Y. J. Lee Y. I. (2019). The impairments of alpha-synuclein and mechanistic target of rapamycin in rotenone-induced SH-SY5Y cells and mice model of parkinson's disease. Front. Neurosci. 13:1028. 10.3389/fnins.2019.01028 31611767 PMC6769080

[B147] Rani L. Mondal A. C. (2020). Emerging concepts of mitochondrial dysfunction in Parkinson's disease progression: Pathogenic and therapeutic implications. Mitochondrion 50, 25–34. 10.1016/j.mito.2019.09.010 31654753

[B148] Ravanidis S. Bougea A. Papagiannakis N. Maniati M. Koros C. Simitsi A. M.. (2020). Circulating Brain-enriched MicroRNAs for detection and discrimination of idiopathic and genetic Parkinson's disease. Mov. Disord. 35, 457–467. 10.1002/mds.27928 31799764

[B149] Reane D. V. Rizzuto R. Raffaello A. (2020). The ER-mitochondria tether at the hub of Ca^2+^ signaling. Curr. Op. Physiol. 17, 261–268. 10.1016/j.cophys.2020.08.013

[B150] Redensek S. Trost M. Dolzan V. (2017). Genetic determinants of parkinson's disease: can they help to stratify the patients based on the underlying molecular defect? Front. Aging Neurosci. 9:20. 10.3389/fnagi.2017.00020 28239348 PMC5301007

[B151] Reinhart F. El Massri N. Johnstone D. M. Stone J. Mitrofanis J. Benabid A. L.. (2016a). Near-infrared light (670 nm) reduces MPTP-induced parkinsonism within a broad therapeutic time window. Exp. Brain Res. 234, 1787–1794. 10.1007/s00221-016-4578-8 26879772

[B152] Reinhart F. Massri N. E. Chabrol C. Cretallaz C. Johnstone D. M. Torres N.. (2016b). Intracranial application of near-infrared light in a hemi-parkinsonian rat model: the impact on behavior and cell survival. J. Neurosurg. 124, 1829–1841. 10.3171/2015.5.JNS15735 26613166

[B153] Reinhart F. Massri N. E. Darlot F. Torres N. Johnstone D. M. Chabrol C.. (2015). 810nm near-infrared light offers neuroprotection and improves locomotor activity in MPTP-treated mice. Neurosci. Res. 92, 86–90. 10.1016/j.neures.2014.11.005 25462595

[B154] Reinhart F. Massri N. E. Torres N. Chabrol C. Molet J. Johnstone D. M.. (2017). The behavioural and neuroprotective outcomes when 670nm and 810nm near infrared light are applied together in MPTP-treated mice. Neurosci. Res. 117, 42–47. 10.1016/j.neures.2016.11.006 27871905

[B155] Rezaee Z. Marandi S. M. Alaei H. Esfarjani F. Feyzollahzadeh S. (2019). Effects of preventive treadmill exercise on the recovery of metabolic and mitochondrial factors in the 6-hydroxydopamine rat model of parkinson's disease. Neurotox. Res. 35, 908–917. 10.1007/s12640-019-0004-x 30820889

[B156] Rubio-Osornio M. Orozco-Ibarra M. Diaz-Ruiz A. Brambila E. Boll M. C. Monroy-Noyola A.. (2017). Copper sulfate pretreatment prevents mitochondrial electron transport chain damage and apoptosis against MPP(+)-induced neurotoxicity. Chem. Biol. Interact. 271, 1–8. 10.1016/j.cbi.2017.04.016 28442376

[B157] Sabouny R. Shutt T. E. (2020). Reciprocal regulation of mitochondrial fission and fusion. Trends Biochem. Sci. 45, 564–577. 10.1016/j.tibs.2020.03.009 32291139

[B158] Salehpour F. Hamblin M. R. (2020). Photobiomodulation for Parkinson's disease in animal models: a systematic review. Biomolecules 10:610. 10.3390/biom10040610 32326425 PMC7225948

[B159] Salehpour F. Mahmoudi J. Kamari F. Sadigh-Eteghad S. Rasta S. H. Hamblin M. R. (2018). Brain photobiomodulation therapy: a narrative review. Mol. Neurobiol. 55, 6601–6636. 10.1007/s12035-017-0852-4 29327206 PMC6041198

[B160] Santos L. Olmo-Aguado S. D. Valenzuela P. L. Winge K. Iglesias-Soler E. Arguelles-Luis J.. (2019). Photobiomodulation in Parkinson's disease: a randomized controlled trial. Brain Stimul. 12, 810–812. 10.1016/j.brs.2019.02.009 30824206

[B161] Sathe A. G. Tuite P. Chen C. Ma Y. Chen W. Cloyd J.. (2020). Pharmacokinetics, safety, and tolerability of orally administered ursodeoxycholic acid in patients with parkinson's disease-a pilot study. J. Clin. Pharmacol. 60, 744–750. 10.1002/jcph.1575 32052462 PMC7245554

[B162] Schulz J. Takousis P. Wohlers I. Itua I. O. G. Dobricic V. Rucker G.. (2019). Meta-analyses identify differentially expressed micrornas in Parkinson's disease. Ann. Neurol. 85, 835–851. 10.1002/ana.25490 30990912

[B163] Seager R. Lee L. Henley J. M. Wilkinson K. A. (2020). Mechanisms and roles of mitochondrial localisation and dynamics in neuronal function. Neuronal. Signal. 4:NS20200008. 10.1042/NS20200008 32714603 PMC7373250

[B164] Shanmughapriya S. Langford D. Natarajaseenivasan K. (2020). Inter and intracellular mitochondrial trafficking in health and disease. Ageing Res. Rev. 62:101128. 10.1016/j.arr.2020.101128 32712108 PMC7484258

[B165] Shaw V. E. Peoples C. Spana S. Ashkan K. Benabid A. L. Stone J.. (2012). Patterns of cell activity in the subthalamic region associated with the neuroprotective action of near-infrared light treatment in MPTP-treated mice. Parkinsons. Dis. 2012:296875. 10.1155/2012/296875 22666627 PMC3361324

[B166] Shi X. Zhao M. Fu C. Fu A. (2017). Intravenous administration of mitochondria for treating experimental Parkinson's disease. Mitochondrion 34, 91–100. 10.1016/j.mito.2017.02.005 28242362

[B167] Shin E. J. Nam Y. Lee J. W. Nguyen P. T. Yoo J. E. Tran T. V.. (2016). N-Methyl, N-propynyl-2-phenylethylamine (MPPE), a selegiline Analog, attenuates mptp-induced dopaminergic toxicity with guaranteed behavioral safety: involvement of inhibitions of mitochondrial oxidative burdens and p53 gene-elicited pro-apoptotic change. Mol. Neurobiol. 53, 6251–6269. 10.1007/s12035-015-9527-1 26563498

[B168] Silveira P. C. L. Ferreira G. K. Zaccaron R. P. Glaser V. Remor A. P. Mendes C.. (2019). Effects of photobiomodulation on mitochondria of brain, muscle, and C6 astroglioma cells. Med. Eng. Phys. 71, 108–113. 10.1016/j.medengphy.2019.05.008 31303375

[B169] Singh A. Kukreti R. Saso L. Kukreti S. (2019). Oxidative stress: a key modulator in neurodegenerative diseases. Molecules 24:1583. 10.3390/molecules24081583 31013638 PMC6514564

[B170] Smith G. M. Gallo G. (2018). The role of mitochondria in axon development and regeneration. Dev. Neurobiol. 78, 221–237. 10.1002/dneu.22546 29030922 PMC5816701

[B171] Sun F. Deng Y. Han X. Liu Q. Zhang P. Manzoor R.. (2019). A secret that underlies Parkinson's disease: the damaging cycle. Neurochem. Int. 129:104484. 10.1016/j.neuint.2019.104484 31173779

[B172] Takeda K. Yanagi S. (2019). Mitochondrial retrograde signaling to the endoplasmic-reticulum regulates unfolded protein responses. Mol. Cell Oncol. 6:e1659078. 10.1080/23723556.2019.1659078 31692879 PMC6816402

[B173] Tang B. L. (2017). Sirtuins as modifiers of Parkinson's disease pathology. J. Neurosci. Res. 95, 930–942. 10.1002/jnr.23806 27402490

[B174] Tarohda T. Ishida Y. Kawai K. Yamamoto M. Amano R. (2005). Regional distributions of manganese, iron, copper, and zinc in the brains of 6-hydroxydopamine-induced parkinsonian rats. Anal. Bioanal. Chem. 383, 224–234. 10.1007/s00216-005-3423-x 16132122

[B175] Titova N. Chaudhuri K. R. (2017). Personalized medicine in Parkinson's disease: time to be precise. Mov. Disord. 32, 1147–1154. 10.1002/mds.27027 28605054 PMC5575483

[B176] Titze-de-Almeida S. S. Soto-Sanchez C. Fernandez E. Koprich J. B. Brotchie J. M. Titze-De-Almeida R. (2020). The promise and challenges of developing miRNA-based therapeutics for parkinson's disease. Cells 9:841. 10.3390/cells9040841 32244357 PMC7226753

[B177] Todd A. M. Staveley B. E. (2008). Pink1 suppresses alpha-synuclein-induced phenotypes in a drosophila model of Parkinson's disease. Genome 51, 1040–1046. 10.1139/G08-085 19088817

[B178] Toyofuku T. Okamoto Y. Ishikawa T. Sasawatari S. Kumanogoh A. (2020). LRRK2 regulates endoplasmic reticulum-mitochondrial tethering through the PERK-mediated ubiquitination pathway. EMBO J. 39:e100875. 10.15252/embj.2020105826 31821596 PMC6960452

[B179] Valdinocci D. Simoes R. F. Kovarova J. Cunha-Oliveira T. Neuzil J. Pountney D. L. (2019). Intracellular and intercellular mitochondrial dynamics in parkinson's disease. Front. Neurosci 13:930. 10.3389/fnins.2019.00930 31619944 PMC6760022

[B180] Valente E. M. Abou-Sleiman P. M. Caputo V. Muqit M. M. Harvey K. Gispert S.. (2004). Hereditary early-onset Parkinson's disease caused by mutations in PINK1. Science 304, 1158–1160. 10.1126/science.1096284 15087508

[B181] Vos M. Esposito G. Edirisinghe J. N. Vilain S. Haddad D. M. Slabbaert J. R.. (2012). Vitamin K2 is a mitochondrial electron carrier that rescues pink1 deficiency. Science 336, 1306–1310. 10.1126/science.1218632 22582012

[B182] Wai T. Langer T. (2016). Mitochondrial dynamics and metabolic regulation. Trends Endocrinol. Metab. 27, 105–117. 10.1016/j.tem.2015.12.001 26754340

[B183] Wallings R. L. Herrick M. K. Tansey M. G. (2020). Linking mitochondria to the immune response. Elife 9:e56214. 10.7554/eLife.56214 32293561 PMC7159921

[B184] Wang D. Xu L. Lv L. Su L. Y. Fan Y. Zhang D. F.. (2015). Association of the LRRK2 genetic polymorphisms with leprosy in Han Chinese from Southwest China. Genes Immun. 16, 112–119. 10.1038/gene.2014.72 25521227

[B185] Wang S. Zhang J. Deng X. Zhao Y. Xu K. (2020). Advances in characterization of SIRT3 deacetylation targets in mitochondrial function. Biochimie 179, 1–13. 10.1016/j.biochi.2020.08.021 32898647

[B186] Wang Y. He J. Liao M. Hu M. Li W. Ouyang H.. (2019). An overview of Sirtuins as potential therapeutic target: structure, function and modulators. Eur. J. Med. Chem. 161, 48–77. 10.1016/j.ejmech.2018.10.028 30342425

[B187] Wang Z. Arat S. Magid-Slav M. Brown J. R. (2018). Meta-analysis of human gene expression in response to mycobacterium tuberculosis infection reveals potential therapeutic targets. BMC Syst. Biol. 12:3. 10.1186/s12918-017-0524-z 29321020 PMC5763539

[B188] Wang Z. B. Liu J. Y. Xu X. J. Mao X. Y. Zhang W. Zhou H. H.. (2019). Neurodegeneration with brain iron accumulation: insights into the mitochondria dysregulation. Biomed. Pharmacother. 118:109068. 10.1016/j.biopha.2019.109068 31404774

[B189] Weindel C. G. Bell S. L. Vail K. J. West K. O. Patrick K. L. Watson R. O. (2020). LRRK2 maintains mitochondrial homeostasis and regulates innate immune responses to Mycobacterium tuberculosis. Elife 9:e51071. 10.7554/eLife.51071.sa2 32057291 PMC7159881

[B190] Whitley B. N. Engelhart E. A. Hoppins S. (2019). Mitochondrial dynamics and their potential as a therapeutic target. Mitochondrion 49, 269–283. 10.1016/j.mito.2019.06.002 31228566 PMC6885535

[B191] Wilson H. Pagano G. De Natale E. R. Mansur A. Caminiti S. P. Polychronis S.. (2020). Mitochondrial Complex 1, Sigma 1, and Synaptic Vesicle 2A in early drug-naive parkinson's disease. Mov. Disord. 35, 1416–1427. 10.1002/mds.28064 32347983

[B192] Yamaguchi A. Ishikawa K. I. Inoshita T. Shiba-Fukushima K. Saiki S. Hatano T.. (2020). Identifying therapeutic agents for amelioration of mitochondrial clearance disorder in neurons of familial Parkinson disease. Stem Cell Rep. 14, 1060–1075. 10.1016/j.stemcr.2020.04.011 32470327 PMC7355139

[B193] Yang L. Youngblood H. Wu C. Zhang Q. (2020). Mitochondria as a target for neuroprotection: role of methylene blue and photobiomodulation. Transl. Neurodegener. 9:19. 10.1186/s40035-020-00197-z 32475349 PMC7262767

[B194] Yang X. Zhang Y. Xu H. Luo X. Yu J. Liu J.. (2016). Neuroprotection of coenzyme Q10 in neurodegenerative diseases. Curr. Top. Med. Chem. 16, 858–866. 10.2174/1568026615666150827095252 26311425

[B195] Yang Y. Gehrke S. Imai Y. Huang Z. Ouyang Y. Wang J. W.. (2006). Mitochondrial pathology and muscle and dopaminergic neuron degeneration caused by inactivation of drosophila pink1 is rescued by Parkin. Proc. Natl. Acad. Sci. U.S.A. 103, 10793–10798. 10.1073/pnas.0602493103 16818890 PMC1502310

[B196] Yasuda T. Hayakawa H. Nihira T. Ren Y. R. Nakata Y. Nagai M.. (2011). Parkin-mediated protection of dopaminergic neurons in a chronic MPTP-minipump mouse model of Parkinson disease. J. Neuropathol. Exp. Neurol. 70, 686–697. 10.1097/NEN.0b013e3182269ecd 21760537

[B197] Zhang F. R. Huang W. Chen S. M. Sun L. D. Liu H. Li Y.. (2009). Genomewide association study of leprosy. N. Engl. J. Med. 361, 2609–2618. 10.1056/NEJMoa0903753 20018961

[B198] Zhang X. Du L. Zhang W. Yang Y. Zhou Q. Du G. (2017). Therapeutic effects of baicalein on rotenone-induced Parkinson's disease through protecting mitochondrial function and biogenesis. Sci. Rep. 7:9968. 10.1038/s41598-017-07442-y 28855526 PMC5577282

[B199] Zhang Y. Anoopkumar-Dukie S. Arora D. Davey A. K. (2020). Review of the anti-inflammatory effect of SIRT1 and SIRT2 modulators on neurodegenerative diseases. Eur. J. Pharmacol. 867:172847. 10.1016/j.ejphar.2019.172847 31812544

[B200] Zhu Z. G. Sun M. X. Zhang W. L. Wang W. W. Jin Y. M. Xie C. L. (2017). The efficacy and safety of coenzyme Q10 in Parkinson's disease: a meta-analysis of randomized controlled trials. Neurol. Sci. 38, 215–224. 10.1007/s10072-016-2757-9 27830343

