# Supplementary material for: Targeting Mitochondrial Impairment in Parkinson's Disease: Challenges and Opportunities
Source: Front Cell Dev Biol. 2021 Jan 5;8:615461. doi: 10.3389/fcell.2020.615461 (PMC7813753; doi:10.3389/fcell.2020.615461)
Supplement: Supplementary file 1 [file Table_1.docx]

Supplementary Table 1. Monogenic causes of PD and association with mitochondrial dysfunction

| Gene name | Genomic coordinates (GRCh38) | Parkinson’s loci designation | MDSGene Designation (PMID 30584170) | Mode of inheritance | Parkinson’s disease phenotype | Mitochondrial involvement in disease pathophysiology – key mechanisms | References |
| --- | --- | --- | --- | --- | --- | --- | --- |
| *SNCA* | 4:89,724,098-89,838,323 | PARK1 | PARK-*SNCA* | AD | May be atypical (cognitive and psychiatric features more likely) | Mitochondrial toxicity, fragmented mitochondria | (Polymeropoulos et al., 1997); (Singleton et al., 2003);(Chartier-Harlin et al., 2004) |
| *PRKN* | 6:161,347,416-162,727,801 | PARK2 | PARK-*Parkin* | AR | Early-onset PD | Defective mitochondrial quality control | (Kitada et al., 1998);(Ge et al., 2020) |
| *PINK1* | 1:20,633,457-20,651,510 | PARK6 | PARK-*PINK1* | AR | Early-onset PD | Defective mitochondrial quality control | (Valente et al., 2004);(Ge et al., 2020) |
| *DJ1* | 1:7,961,710-7,985,504 | PARK7 | PARK-*DJ1* | AR | Early-onset PD | Reduced anti-oxidative stress activities | (Bonifati et al., 2003);(Takahashi-Niki et al., 2004) |
| *LRRK2* | 12:40,224,889-40,369,284 | PARK8 | PARK-*LRRK2* | AD | Typical PD/resembling IPD | Disturbance in mitochondrial ATP and reactive oxygen species production, mitochondrial fusion and fission, mitophagy, mitochondrial DNA damage, calcium homeostasis | (Zimprich et al., 2004);(Mancini et al., 2020) |
| *ATP13A2* | 1:16,985,957-17,011,971 | PARK9 | PARK-*ATP13A2* | AR | Atypical PD/Kufor-Rakeb syndrome | Impaired mitochondrial clearance, mitochondrial dysfunction due to zinc dyshomeostasis | (Ramirez et al., 2006);(Grunewald et al., 2012);(Park et al., 2014) |
| *HTRA2** | 2:74,529,404-74,533,555 | PARK13 | - | AD | Typical PD/resembling IPD | Altered mitochondrial morphology, susceptibility to stress induced cell death | (Strauss et al., 2005) |
| *PLA2G6* | 22:38,111,494-38,181,829 | PARK14 | DYT/PARK-*PLA2G6* | AR | Atypical PD/Neurodegeneration with brain iron accumulation type 2B/Infantile neuroaxonal dystrophy 1 | Maintenance of mitochondrial function/impaired mitophagy | (Paisan-Ruiz et al., 2009);(Chiu et al., 2017);(Chiu et al., 2019) |
| *FBXO7* | 22:32,474,681-32,498,828 | PARK15 | PARK-*FBXO7* | AR | Atypical PD | Aggravated protein aggregation in mitochondria, impaired mitophagy | (Shojaee et al., 2008);(Zhou et al., 2018) |
| *VPS35* | 16:46,656,131-46,689,193 | PARK17 | PARK-*VPS35* | AD | Typical PD/resembling IPD | Regulation of mitochondrial dynamics and homeostasis | (Vilarino-Guell et al., 2011);(Zimprich et al., 2011);(Cutillo et al., 2020) |
| *EIF4G1** | 3:184,314,605-184,335,357 | PARK18 | - | AD | Typical PD, Lewy body disease | Loss of mitochondrial membrane potential | (Chartier-Harlin et al., 2011) |
| *DNAJC6** | 1:65,264,748-65,415,870 | PARK19A, PARK19B | PARK-*DNAJC6* | AR | Juvenile onset (PARK19A), early onset PD (PARK19B) | - | (Edvardson et al., 2012) |
| *SYNJ1** | 21:32,628,758-32,731,246 | PARK20 | PARK-*SYNJ1* | AR | Atypical early onset PD | - | (Quadri et al., 2013) |
| *DNAJC13** | 3:132,417,501-132,539,031 | PARK21 | - | AD | Typical | - | (Vilarino-Guell et al., 2014) |
| *CHCHD2** | 7:56,101,572-56,106,475 | PARK22 | PARK-*CHCHD2* | AD | Typical | Impaired regulation of the role of cytochrome c in oxidative phosphorylation and cell death in response to mitochondrial stress | (Funayama et al., 2015);(Meng et al., 2017) |
| *VPS13C** | 15:61,852,388-62,060,446 | PARK23 | PARK-*VPS13C* | AR | Atypical early onset PD | Numerous effects on mitochondrial function including Increased PINK1/Parkin-dependent mitophagy | (Lesage et al., 2016) |
| *LRP10** | 14:22,871,612-22,881,712 | - | - | AD | Typical PD, PD dementia, dementia with Lewy bodies | - | (Quadri et al., 2018) |
| *GBA* | 1:155,234,451-155,244,626 | - | PARK-*GBA* | AD | Typical PD with more rapid cognitive and motor progression, dementia with Lewy bodies | Impaired mitophagy | (Sidransky et al., 2009;Barkhuizen et al., 2016;Zhao et al., 2016);(Gegg and Schapira, 2016);(Moren et al., 2019) |
| *DCTN1* | 2:74,361,154-74,391,865 | - | PARK-*DCTN1* | AD | Perry syndrome | - | (Farrer et al., 2009) |
| *CSMD1** | 8:2,935,352-4,995,034 | - | - | AD | Typical PD/resembling IPD | - | (Ruiz-Martinez et al., 2017) |
| *ACMSD** | 2:134,838,616-134,902,034 | - | - | AD/sporadic | Typical PD/resembling IPD | May lead to an accumulation of quinolinate causing mitochondrial dysfunction | (Vilas et al., 2017) |
| *NUS1** | 6:117,675,468-117,710,726 | - | - | De novo dominant | Early-onset PD |  | (Guo et al., 2018) |

AD, autosomal dominant; AR, autosomal recessive; IPD, Idiopathic Parkinson’s disease; PD, Parkinson’s disease

# PARK loci not numerically represented have not been confirmed and no gene has been found since they were first reported, likely representing a risk factor and not a monogenic cause, * Recently described/requires further investigation and validation

**References**

Barkhuizen, M., Anderson, D.G., and Grobler, A.F. (2016). Advances in GBA-associated Parkinson's disease--Pathology, presentation and therapies. *Neurochem Int* 93**,** 6-25. doi:10.1016/j.neuint.2015.12.004

Bonifati, V., Rizzu, P., Van Baren, M.J., Schaap, O., Breedveld, G.J., Krieger, E., Dekker, M.C., Squitieri, F., Ibanez, P., Joosse, M., Van Dongen, J.W., Vanacore, N., Van Swieten, J.C., Brice, A., Meco, G., Van Duijn, C.M., Oostra, B.A., and Heutink, P. (2003). Mutations in the DJ-1 gene associated with autosomal recessive early-onset parkinsonism. *Science* 299**,** 256-259. doi:10.1126/science.1077209

Chartier-Harlin, M.C., Dachsel, J.C., Vilarino-Guell, C., Lincoln, S.J., Lepretre, F., Hulihan, M.M., Kachergus, J., Milnerwood, A.J., Tapia, L., Song, M.S., Le Rhun, E., Mutez, E., Larvor, L., Duflot, A., Vanbesien-Mailliot, C., Kreisler, A., Ross, O.A., Nishioka, K., Soto-Ortolaza, A.I., Cobb, S.A., Melrose, H.L., Behrouz, B., Keeling, B.H., Bacon, J.A., Hentati, E., Williams, L., Yanagiya, A., Sonenberg, N., Lockhart, P.J., Zubair, A.C., Uitti, R.J., Aasly, J.O., Krygowska-Wajs, A., Opala, G., Wszolek, Z.K., Frigerio, R., Maraganore, D.M., Gosal, D., Lynch, T., Hutchinson, M., Bentivoglio, A.R., Valente, E.M., Nichols, W.C., Pankratz, N., Foroud, T., Gibson, R.A., Hentati, F., Dickson, D.W., Destee, A., and Farrer, M.J. (2011). Translation initiator EIF4G1 mutations in familial Parkinson disease. *Am J Hum Genet* 89**,** 398-406. doi:10.1016/j.ajhg.2011.08.009

Chartier-Harlin, M.C., Kachergus, J., Roumier, C., Mouroux, V., Douay, X., Lincoln, S., Levecque, C., Larvor, L., Andrieux, J., Hulihan, M., Waucquier, N., Defebvre, L., Amouyel, P., Farrer, M., and Destee, A. (2004). Alpha-synuclein locus duplication as a cause of familial Parkinson's disease. *Lancet* 364**,** 1167-1169. doi:10.1016/S0140-6736(04)17103-1

Chiu, C.C., Lu, C.S., Weng, Y.H., Chen, Y.L., Huang, Y.Z., Chen, R.S., Cheng, Y.C., Huang, Y.C., Liu, Y.C., Lai, S.C., Lin, K.J., Lin, Y.W., Chen, Y.J., Chen, C.L., Yeh, T.H., and Wang, H.L. (2019). PARK14 (D331Y) PLA2G6 Causes Early-Onset Degeneration of Substantia Nigra Dopaminergic Neurons by Inducing Mitochondrial Dysfunction, ER Stress, Mitophagy Impairment and Transcriptional Dysregulation in a Knockin Mouse Model. *Mol Neurobiol* 56**,** 3835-3853. doi:10.1007/s12035-018-1118-5

Chiu, C.C., Yeh, T.H., Lu, C.S., Huang, Y.C., Cheng, Y.C., Huang, Y.Z., Weng, Y.H., Liu, Y.C., Lai, S.C., Chen, Y.L., Chen, Y.J., Chen, C.L., Chen, H.Y., Lin, Y.W., and Wang, H.L. (2017). PARK14 PLA2G6 mutants are defective in preventing rotenone-induced mitochondrial dysfunction, ROS generation and activation of mitochondrial apoptotic pathway. *Oncotarget* 8**,** 79046-79060. doi:10.18632/oncotarget.20893

Cutillo, G., Simon, D.K., and Eleuteri, S. (2020). VPS35 and the mitochondria: Connecting the dots in Parkinson's disease pathophysiology. *Neurobiol Dis* 145**,** 105056. doi:10.1016/j.nbd.2020.105056

Edvardson, S., Cinnamon, Y., Ta-Shma, A., Shaag, A., Yim, Y.I., Zenvirt, S., Jalas, C., Lesage, S., Brice, A., Taraboulos, A., Kaestner, K.H., Greene, L.E., and Elpeleg, O. (2012). A deleterious mutation in DNAJC6 encoding the neuronal-specific clathrin-uncoating co-chaperone auxilin, is associated with juvenile parkinsonism. *PLoS One* 7**,** e36458. doi:10.1371/journal.pone.0036458

Farrer, M.J., Hulihan, M.M., Kachergus, J.M., Dachsel, J.C., Stoessl, A.J., Grantier, L.L., Calne, S., Calne, D.B., Lechevalier, B., Chapon, F., Tsuboi, Y., Yamada, T., Gutmann, L., Elibol, B., Bhatia, K.P., Wider, C., Vilarino-Guell, C., Ross, O.A., Brown, L.A., Castanedes-Casey, M., Dickson, D.W., and Wszolek, Z.K. (2009). DCTN1 mutations in Perry syndrome. *Nat Genet* 41**,** 163-165. doi:10.1038/ng.293

Funayama, M., Ohe, K., Amo, T., Furuya, N., Yamaguchi, J., Saiki, S., Li, Y., Ogaki, K., Ando, M., Yoshino, H., Tomiyama, H., Nishioka, K., Hasegawa, K., Saiki, H., Satake, W., Mogushi, K., Sasaki, R., Kokubo, Y., Kuzuhara, S., Toda, T., Mizuno, Y., Uchiyama, Y., Ohno, K., and Hattori, N. (2015). CHCHD2 mutations in autosomal dominant late-onset Parkinson's disease: a genome-wide linkage and sequencing study. *Lancet Neurol* 14**,** 274-282. doi:10.1016/S1474-4422(14)70266-2

Ge, P., Dawson, V.L., and Dawson, T.M. (2020). PINK1 and Parkin mitochondrial quality control: a source of regional vulnerability in Parkinson's disease. *Mol Neurodegener* 15**,** 20. doi:10.1186/s13024-020-00367-7

Gegg, M.E., and Schapira, A.H. (2016). Mitochondrial dysfunction associated with glucocerebrosidase deficiency. *Neurobiol Dis* 90**,** 43-50. doi:10.1016/j.nbd.2015.09.006

Grunewald, A., Arns, B., Seibler, P., Rakovic, A., Munchau, A., Ramirez, A., Sue, C.M., and Klein, C. (2012). ATP13A2 mutations impair mitochondrial function in fibroblasts from patients with Kufor-Rakeb syndrome. *Neurobiol Aging* 33**,** 1843 e1841-1847. doi:10.1016/j.neurobiolaging.2011.12.035

Guo, J.F., Zhang, L., Li, K., Mei, J.P., Xue, J., Chen, J., Tang, X., Shen, L., Jiang, H., Chen, C., Guo, H., Wu, X.L., Sun, S.L., Xu, Q., Sun, Q.Y., Chan, P., Shang, H.F., Wang, T., Zhao, G.H., Liu, J.Y., Xie, X.F., Jiang, Y.Q., Liu, Z.H., Zhao, Y.W., Zhu, Z.B., Li, J.D., Hu, Z.M., Yan, X.X., Fang, X.D., Wang, G.H., Zhang, F.Y., Xia, K., Liu, C.Y., Zhu, X.W., Yue, Z.Y., Li, S.C., Cai, H.B., Zhang, Z.H., Duan, R.H., and Tang, B.S. (2018). Coding mutations in NUS1 contribute to Parkinson's disease. *Proc Natl Acad Sci U S A* 115**,** 11567-11572. doi:10.1073/pnas.1809969115

Kitada, T., Asakawa, S., Hattori, N., Matsumine, H., Yamamura, Y., Minoshima, S., Yokochi, M., Mizuno, Y., and Shimizu, N. (1998). Mutations in the parkin gene cause autosomal recessive juvenile parkinsonism. *Nature* 392**,** 605-608. doi:10.1038/33416

Lesage, S., Drouet, V., Majounie, E., Deramecourt, V., Jacoupy, M., Nicolas, A., Cormier-Dequaire, F., Hassoun, S.M., Pujol, C., Ciura, S., Erpapazoglou, Z., Usenko, T., Maurage, C.A., Sahbatou, M., Liebau, S., Ding, J., Bilgic, B., Emre, M., Erginel-Unaltuna, N., Guven, G., Tison, F., Tranchant, C., Vidailhet, M., Corvol, J.C., Krack, P., Leutenegger, A.L., Nalls, M.A., Hernandez, D.G., Heutink, P., Gibbs, J.R., Hardy, J., Wood, N.W., Gasser, T., Durr, A., Deleuze, J.F., Tazir, M., Destee, A., Lohmann, E., Kabashi, E., Singleton, A., Corti, O., Brice, A., French Parkinson's Disease Genetics, S., and International Parkinson's Disease Genomics, C. (2016). Loss of VPS13C Function in Autosomal-Recessive Parkinsonism Causes Mitochondrial Dysfunction and Increases PINK1/Parkin-Dependent Mitophagy. *Am J Hum Genet* 98**,** 500-513. doi:10.1016/j.ajhg.2016.01.014

Mancini, A., Mazzocchetti, P., Sciaccaluga, M., Megaro, A., Bellingacci, L., Beccano-Kelly, D.A., Di Filippo, M., Tozzi, A., and Calabresi, P. (2020). From Synaptic Dysfunction to Neuroprotective Strategies in Genetic Parkinson's Disease: Lessons From LRRK2. *Front Cell Neurosci* 14**,** 158. doi:10.3389/fncel.2020.00158

Meng, H., Yamashita, C., Shiba-Fukushima, K., Inoshita, T., Funayama, M., Sato, S., Hatta, T., Natsume, T., Umitsu, M., Takagi, J., Imai, Y., and Hattori, N. (2017). Loss of Parkinson's disease-associated protein CHCHD2 affects mitochondrial crista structure and destabilizes cytochrome c. *Nat Commun* 8**,** 15500. doi:10.1038/ncomms15500

Moren, C., Juarez-Flores, D.L., Chau, K.Y., Gegg, M., Garrabou, G., Gonzalez-Casacuberta, I., Guitart-Mampel, M., Tolosa, E., Marti, M.J., Cardellach, F., and Schapira, A.H.V. (2019). GBA mutation promotes early mitochondrial dysfunction in 3D neurosphere models. *Aging (Albany NY)* 11**,** 10338-10355. doi:10.18632/aging.102460

Paisan-Ruiz, C., Bhatia, K.P., Li, A., Hernandez, D., Davis, M., Wood, N.W., Hardy, J., Houlden, H., Singleton, A., and Schneider, S.A. (2009). Characterization of PLA2G6 as a locus for dystonia-parkinsonism. *Ann Neurol* 65**,** 19-23. doi:10.1002/ana.21415

Park, J.S., Koentjoro, B., Veivers, D., Mackay-Sim, A., and Sue, C.M. (2014). Parkinson's disease-associated human ATP13A2 (PARK9) deficiency causes zinc dyshomeostasis and mitochondrial dysfunction. *Hum Mol Genet* 23**,** 2802-2815. doi:10.1093/hmg/ddt623

Polymeropoulos, M.H., Lavedan, C., Leroy, E., Ide, S.E., Dehejia, A., Dutra, A., Pike, B., Root, H., Rubenstein, J., Boyer, R., Stenroos, E.S., Chandrasekharappa, S., Athanassiadou, A., Papapetropoulos, T., Johnson, W.G., Lazzarini, A.M., Duvoisin, R.C., Di Iorio, G., Golbe, L.I., and Nussbaum, R.L. (1997). Mutation in the alpha-synuclein gene identified in families with Parkinson's disease. *Science* 276**,** 2045-2047. doi:10.1126/science.276.5321.2045

Quadri, M., Fang, M., Picillo, M., Olgiati, S., Breedveld, G.J., Graafland, J., Wu, B., Xu, F., Erro, R., Amboni, M., Pappata, S., Quarantelli, M., Annesi, G., Quattrone, A., Chien, H.F., Barbosa, E.R., International Parkinsonism Genetics, N., Oostra, B.A., Barone, P., Wang, J., and Bonifati, V. (2013). Mutation in the SYNJ1 gene associated with autosomal recessive, early-onset Parkinsonism. *Hum Mutat* 34**,** 1208-1215. doi:10.1002/humu.22373

Quadri, M., Mandemakers, W., Grochowska, M.M., Masius, R., Geut, H., Fabrizio, E., Breedveld, G.J., Kuipers, D., Minneboo, M., Vergouw, L.J.M., Carreras Mascaro, A., Yonova-Doing, E., Simons, E., Zhao, T., Di Fonzo, A.B., Chang, H.C., Parchi, P., Melis, M., Correia Guedes, L., Criscuolo, C., Thomas, A., Brouwer, R.W.W., Heijsman, D., Ingrassia, A.M.T., Calandra Buonaura, G., Rood, J.P., Capellari, S., Rozemuller, A.J., Sarchioto, M., Fen Chien, H., Vanacore, N., Olgiati, S., Wu-Chou, Y.H., Yeh, T.H., Boon, A.J.W., Hoogers, S.E., Ghazvini, M., As, I.J., Van, I.W.F.J., Onofrj, M., Barone, P., Nicholl, D.J., Puschmann, A., De Mari, M., Kievit, A.J., Barbosa, E., De Michele, G., Majoor-Krakauer, D., Van Swieten, J.C., De Jong, F.J., Ferreira, J.J., Cossu, G., Lu, C.S., Meco, G., Cortelli, P., Van De Berg, W.D.J., Bonifati, V., and International Parkinsonism Genetics, N. (2018). LRP10 genetic variants in familial Parkinson's disease and dementia with Lewy bodies: a genome-wide linkage and sequencing study. *Lancet Neurol* 17**,** 597-608. doi:10.1016/S1474-4422(18)30179-0

Ramirez, A., Heimbach, A., Grundemann, J., Stiller, B., Hampshire, D., Cid, L.P., Goebel, I., Mubaidin, A.F., Wriekat, A.L., Roeper, J., Al-Din, A., Hillmer, A.M., Karsak, M., Liss, B., Woods, C.G., Behrens, M.I., and Kubisch, C. (2006). Hereditary parkinsonism with dementia is caused by mutations in ATP13A2, encoding a lysosomal type 5 P-type ATPase. *Nat Genet* 38**,** 1184-1191. doi:10.1038/ng1884

Ruiz-Martinez, J., Azcona, L.J., Bergareche, A., Marti-Masso, J.F., and Paisan-Ruiz, C. (2017). Whole-exome sequencing associates novel CSMD1 gene mutations with familial Parkinson disease. *Neurol Genet* 3**,** e177. doi:10.1212/NXG.0000000000000177

Shojaee, S., Sina, F., Banihosseini, S.S., Kazemi, M.H., Kalhor, R., Shahidi, G.A., Fakhrai-Rad, H., Ronaghi, M., and Elahi, E. (2008). Genome-wide linkage analysis of a Parkinsonian-pyramidal syndrome pedigree by 500 K SNP arrays. *Am J Hum Genet* 82**,** 1375-1384. doi:10.1016/j.ajhg.2008.05.005

Sidransky, E., Nalls, M.A., Aasly, J.O., Aharon-Peretz, J., Annesi, G., Barbosa, E.R., Bar-Shira, A., Berg, D., Bras, J., Brice, A., Chen, C.M., Clark, L.N., Condroyer, C., De Marco, E.V., Durr, A., Eblan, M.J., Fahn, S., Farrer, M.J., Fung, H.C., Gan-Or, Z., Gasser, T., Gershoni-Baruch, R., Giladi, N., Griffith, A., Gurevich, T., Januario, C., Kropp, P., Lang, A.E., Lee-Chen, G.J., Lesage, S., Marder, K., Mata, I.F., Mirelman, A., Mitsui, J., Mizuta, I., Nicoletti, G., Oliveira, C., Ottman, R., Orr-Urtreger, A., Pereira, L.V., Quattrone, A., Rogaeva, E., Rolfs, A., Rosenbaum, H., Rozenberg, R., Samii, A., Samaddar, T., Schulte, C., Sharma, M., Singleton, A., Spitz, M., Tan, E.K., Tayebi, N., Toda, T., Troiano, A.R., Tsuji, S., Wittstock, M., Wolfsberg, T.G., Wu, Y.R., Zabetian, C.P., Zhao, Y., and Ziegler, S.G. (2009). Multicenter analysis of glucocerebrosidase mutations in Parkinson's disease. *N Engl J Med* 361**,** 1651-1661. doi:10.1056/NEJMoa0901281

Singleton, A.B., Farrer, M., Johnson, J., Singleton, A., Hague, S., Kachergus, J., Hulihan, M., Peuralinna, T., Dutra, A., Nussbaum, R., Lincoln, S., Crawley, A., Hanson, M., Maraganore, D., Adler, C., Cookson, M.R., Muenter, M., Baptista, M., Miller, D., Blancato, J., Hardy, J., and Gwinn-Hardy, K. (2003). alpha-Synuclein locus triplication causes Parkinson's disease. *Science* 302**,** 841. doi:10.1126/science.1090278

Strauss, K.M., Martins, L.M., Plun-Favreau, H., Marx, F.P., Kautzmann, S., Berg, D., Gasser, T., Wszolek, Z., Muller, T., Bornemann, A., Wolburg, H., Downward, J., Riess, O., Schulz, J.B., and Kruger, R. (2005). Loss of function mutations in the gene encoding Omi/HtrA2 in Parkinson's disease. *Hum Mol Genet* 14**,** 2099-2111. doi:10.1093/hmg/ddi215

Takahashi-Niki, K., Niki, T., Taira, T., Iguchi-Ariga, S.M., and Ariga, H. (2004). Reduced anti-oxidative stress activities of DJ-1 mutants found in Parkinson's disease patients. *Biochem Biophys Res Commun* 320**,** 389-397. doi:10.1016/j.bbrc.2004.05.187

Valente, E.M., Abou-Sleiman, P.M., Caputo, V., Muqit, M.M., Harvey, K., Gispert, S., Ali, Z., Del Turco, D., Bentivoglio, A.R., Healy, D.G., Albanese, A., Nussbaum, R., Gonzalez-Maldonado, R., Deller, T., Salvi, S., Cortelli, P., Gilks, W.P., Latchman, D.S., Harvey, R.J., Dallapiccola, B., Auburger, G., and Wood, N.W. (2004). Hereditary early-onset Parkinson's disease caused by mutations in PINK1. *Science* 304**,** 1158-1160. doi:10.1126/science.1096284

Vilarino-Guell, C., Rajput, A., Milnerwood, A.J., Shah, B., Szu-Tu, C., Trinh, J., Yu, I., Encarnacion, M., Munsie, L.N., Tapia, L., Gustavsson, E.K., Chou, P., Tatarnikov, I., Evans, D.M., Pishotta, F.T., Volta, M., Beccano-Kelly, D., Thompson, C., Lin, M.K., Sherman, H.E., Han, H.J., Guenther, B.L., Wasserman, W.W., Bernard, V., Ross, C.J., Appel-Cresswell, S., Stoessl, A.J., Robinson, C.A., Dickson, D.W., Ross, O.A., Wszolek, Z.K., Aasly, J.O., Wu, R.M., Hentati, F., Gibson, R.A., Mcpherson, P.S., Girard, M., Rajput, M., Rajput, A.H., and Farrer, M.J. (2014). DNAJC13 mutations in Parkinson disease. *Hum Mol Genet* 23**,** 1794-1801. doi:10.1093/hmg/ddt570

Vilarino-Guell, C., Wider, C., Ross, O.A., Dachsel, J.C., Kachergus, J.M., Lincoln, S.J., Soto-Ortolaza, A.I., Cobb, S.A., Wilhoite, G.J., Bacon, J.A., Behrouz, B., Melrose, H.L., Hentati, E., Puschmann, A., Evans, D.M., Conibear, E., Wasserman, W.W., Aasly, J.O., Burkhard, P.R., Djaldetti, R., Ghika, J., Hentati, F., Krygowska-Wajs, A., Lynch, T., Melamed, E., Rajput, A., Rajput, A.H., Solida, A., Wu, R.M., Uitti, R.J., Wszolek, Z.K., Vingerhoets, F., and Farrer, M.J. (2011). VPS35 mutations in Parkinson disease. *Am J Hum Genet* 89**,** 162-167. doi:10.1016/j.ajhg.2011.06.001

Vilas, D., Fernandez-Santiago, R., Sanchez, E., Azcona, L.J., Santos-Montes, M., Casquero, P., Argandona, L., Tolosa, E., and Paisan-Ruiz, C. (2017). A Novel p.Glu298Lys Mutation in the ACMSD Gene in Sporadic Parkinson's Disease. *J Parkinsons Dis* 7**,** 459-463. doi:10.3233/JPD-171146

Zhao, F., Bi, L., Wang, W., Wu, X., Li, Y., Gong, F., Lu, S., Feng, F., Qian, Z., Hu, C., Wu, Y., and Sun, Y. (2016). Mutations of glucocerebrosidase gene and susceptibility to Parkinson's disease: An updated meta-analysis in a European population. *Neuroscience* 320**,** 239-246. doi:10.1016/j.neuroscience.2016.02.007

Zhou, Z.D., Lee, J.C.T., and Tan, E.K. (2018). Pathophysiological mechanisms linking F-box only protein 7 (FBXO7) and Parkinson's disease (PD). *Mutat Res* 778**,** 72-78. doi:10.1016/j.mrrev.2018.10.001

Zimprich, A., Benet-Pages, A., Struhal, W., Graf, E., Eck, S.H., Offman, M.N., Haubenberger, D., Spielberger, S., Schulte, E.C., Lichtner, P., Rossle, S.C., Klopp, N., Wolf, E., Seppi, K., Pirker, W., Presslauer, S., Mollenhauer, B., Katzenschlager, R., Foki, T., Hotzy, C., Reinthaler, E., Harutyunyan, A., Kralovics, R., Peters, A., Zimprich, F., Brucke, T., Poewe, W., Auff, E., Trenkwalder, C., Rost, B., Ransmayr, G., Winkelmann, J., Meitinger, T., and Strom, T.M. (2011). A mutation in VPS35, encoding a subunit of the retromer complex, causes late-onset Parkinson disease. *Am J Hum Genet* 89**,** 168-175. doi:10.1016/j.ajhg.2011.06.008

Zimprich, A., Biskup, S., Leitner, P., Lichtner, P., Farrer, M., Lincoln, S., Kachergus, J., Hulihan, M., Uitti, R.J., Calne, D.B., Stoessl, A.J., Pfeiffer, R.F., Patenge, N., Carbajal, I.C., Vieregge, P., Asmus, F., Muller-Myhsok, B., Dickson, D.W., Meitinger, T., Strom, T.M., Wszolek, Z.K., and Gasser, T. (2004). Mutations in LRRK2 cause autosomal-dominant parkinsonism with pleomorphic pathology. *Neuron* 44**,** 601-607. doi:10.1016/j.neuron.2004.11.005
