# Supplementary material for: Targeting Mitochondrial Impairment in Parkinson's Disease: Challenges and Opportunities
Source: Front Cell Dev Biol. 2021 Jan 5;8:615461. doi: 10.3389/fcell.2020.615461 (PMC7813753; doi:10.3389/fcell.2020.615461)
Supplement: Supplementary file 2 [file Table_2.docx]

Supplementary Table 2. Major clinical trials targeting mitochondrial function in Parkinson’s disease

| **Study publication – abbreviated title** | **Time frame of study** | **Intended effect on mitochondria** | **Study Design** | **Outcome Measure** | **Sample** | **Findings** |
| --- | --- | --- | --- | --- | --- | --- |
| Coenzyme Q10 supplementation provides mild symptomatic benefit in patients with Parkinson's disease (Muller et al., 2003). | 4 weeks | Antioxidant, evidence for preservation of mitochondrial function and neuroprotective effect (Hernandez-Camacho et al., 2018) | Single center, parallel group, placebo controlled, double-blind trial determining effect of coenzyme Q10 | UPDRS and Farnsworth – Munsell 100 Hue test | 28 treated and stable PD | Mild symptomatic benefit and better visual function |
| Symptomatic effects of coenzyme Q(10) in Parkinson disease (Storch et al., 2007). | 3 months | Antioxidant (see above) | Multicenter, randomized, double-blind, placebo-controlled, stratified, parallel-group, single-dose trial on effects of coenzyme Q10 | Primary outcome – change in UPDRS | 131 PD without motor fluctuations on stable treatment | No symptomatic effect |
| High-dosage coenzyme Q10 in early Parkinson disease: no evidence of benefit (Parkinson Study Group et al., 2014). | 16 months | Antioxidant (see above) | Phase III randomized, placebo-controlled, double-blind clinical trial comparing placebo and two doses of coenzyme Q10 | Primary outcome measure being a change in UPDRS score | 600 PD | No clinical benefit |
| Mitochondria-targeted antioxidant MitoQ as a disease-modifying therapy in Parkinson's disease (Snow et al., 2010). | 12 months | Antioxidant, MitoQ has better distribution to mitochondria than coenzyme Q10 | Double-blind, placebo-controlled study comparing MitoQ and placebo | Effect on PD progression measured by UPDRS | 128 newly diagnosed de novo PD | No difference between MitoQ and placebo |
| Reduced coenzyme Q10 for Parkinson's disease (Yoritaka et al., 2015). | 48 or 96 weeks | Antioxidant (the reduced form of CoQ10 (ubiquinol-10) may have stronger neuroprotective effects) | Randomized, double-blind, placebo-controlled, parallel-group pilot trial assessing ubiquinol-10 | UPDRS | PD with wearing off (n =14) and early PD without levodopa (n = 14) | Ubiquinol-10 may improve symptoms for PD with wearing off |
| Creatine supplementation in Parkinson disease (Bender et al., 2006). | 2 years | Creatine and phosphocreatine are critical for ATP homeostasis | Placebo-controlled randomized clinical trial of creatine supplementation | Primary outcome was changes in dopamine transporter SPECT, secondary outcome was disease progression and quality of life | 60 PD | Improved mood, smaller doses of dopaminergic therapy, no effect on UPDRS or dopamine transporter SPECT |
| Treatment of Parkinson disease with diet-induced hyperketonemia: a feasibility study (Vanitallie et al., 2005). | 28 days | Hyperketonemia may bypass impaired complex I activity | Feasibility study, volunteers prepared "hyperketogenic" diet at home | UPDRS | 5 PD | Improvement in UPDRS, unable to exclude placebo effect |
| Exenatide once weekly versus placebo in Parkinson's disease (Athauda et al., 2017). | 48 weeks followed by 12 weeks washout | Exenatide has multiple potential neuroprotective effects including improving mitochondrial biogenesis | Single-centre, randomised, double-blind, placebo-controlled trial comparing exenatide versus placebo | Primary outcome measure – UPDRS | 62 “moderate” PD | Improved off-medication motor scores, sustained beyond period of exposure |
| Aerobic-Strength Exercise Improves Metabolism and Clinical State in Parkinson's Disease Patients (Krumpolec et al., 2017). | 3 months | Exercise has multiple beneficial effects including alleviating mitochondrial dysfunction (see main text). | Endurance/strength training was performed on PD patients and controls and compared with non-exercising PD patients | Outcome measures included resting energy expenditure, glucose metabolism, adiposity, muscle energy metabolism (31P-MRS) and muscle biopsy findings. | 11 early-mid stage PD | Exercise training improved the clinical state, including motor functions and whole-body metabolism. |
| Rasagiline in Parkinson's disease (Olanow et al., 2009). | Rasagiline for 72 weeks or placebo for 36 weeks followed by rasagiline for 36 weeks | Rasagiline may affect mitochondrial function (Jenner and Langston, 2011). Monoamine oxidase B, the symptomatic target of rasagiline, is located in the outer mitochondrial membrane. Furthermore, rasagiline effects numerous mitochondrial mechanisms (Jenner and Langston, 2011). | Double-blind, delayed-start trial of rasagiline | UPDRS | 1176 untreated PD | Rasagiline 1 mg per day provided benefit, 2 mg per day did not. |
| Ambroxol for the Treatment of Patients With Parkinson Disease With and Without Glucocerebrosidase Gene Mutations (Mullin et al., 2020). | Primary outcomes assessed at 186 days | Mitochondria content is increased by ambroxol through the peroxisome proliferator-activated receptor gamma coactivator 1-alpha | Single-center open-label noncontrolled clinical trial | Primary outcome measure - detection of ambroxol in the CSF and a change in CSF glucocerebrosidase activity | 18 PD included in the final analysis | Ambroxol was safe and well tolerated and achieved CSF penetration and target engagement |
| Isradipine Versus Placebo in Early Parkinson Disease (Parkinson Study Group, 2020). | 36 months | Isradipine is a dihydropyridine calcium-channel blocker with neuroprotectant effects including a reduction in mitochondrial oxidative stress. | Multicenter, randomized, parallel-group, double-blind, placebo-controlled trial | Primary outcome being change in UPDRS | 336 early PD | Isradipine did not slow clinical progression. |
| Clinical trials in progress/unpublished# | | | | | | |
| Ursodeoxycholic acid as a novel disease-modifying treatment for Parkinson's disease (Payne et al., 2020). ClinicalTrials.gov Identifier: NCT03840005 | Protocol published 2020. | UDCA is a candidate mitochondrial rescue compound, the mode of action is uncertain but current evidence suggests it is Akt-mediated. | Phase II, two-centre, double-blind, randomised, placebo-controlled trial of UDCA | The primary outcome is safety and tolerability, numerous secondary outcomes. | 30 early PD | - |
| Brain Bioenergetics in Parkinson's Disease and Response to Repeated Oral UDCA Treatment.  ClinicalTrials.gov Identifier: NCT02967250 | - | As above | Open-label phase 1 study | The primary outcomes are plasma UDCA levels and cortical bioenergetic profile and ATPase activity ascertained through MRS. | 20 participants with medically stable mild to moderate Parkinson's disease or healthy controls | - |
| Idebenone Treatment of Early Parkinson's Disease symptoms (ITEP).  ClinicalTrials.gov Identifier: NCT03727295 | - | Idebenone is an analog of CoQ10 but with higher efficacy and a better pharmacokinetic profile (El-Hattab et al., 2017). | Multicenter, parallel, randomized, double-blind, placebo-controlled study | Motor and non-motor symptoms (numerous measures) | 180 participants with early, treated PD | - |
| Study of Urate Elevation in Parkinson's Disease, Phase 3 (SURE-PD3) | - | Urate is a plasma antioxidant, neuroprotective in PD, possibly via the Nrf2 antioxidant response pathway (Crotty et al., 2017;Soderbom, 2020). | Randomized, Double-blind, Placebo-controlled Trial | Rate of clinical decline (MDS-UPDRS) | 298 participants with early PD | - |
| An omics-based strategy using coenzyme  Q10 in patients with Parkinson’s disease | Protocol published 2019 | Antioxidant | Double-blind  randomized placebo-controlled parallel  group trial | Primary endpoint – motor subscore on MDS-UPDRS. Numerous secondary endpoints including magnetic resonance imaging of brain energy metabolism (31P-MRS  imaging) | Subgroups stratified according to variants in mitochondrial genes as follows: biallelic and heterozygous *PRKN*/*PINK1* mutation carriers, “omics” positive and “omics” negative PD patients. | - |
| Nicotinamide Supplementation in Early Parkinson's Disease (NOPARK). ClinicalTrials.gov Identifier: NCT03568968 |  | NAD-deficiency is a key-event in the pathogenesis of PD. Nicotinamide riboside is a precursor NAD and it’s supplementation may correct NAD deficiency and slow progression of PD. | Randomized double-blinded study | MDS-UPDRS | 200 newly diagnosed and/or treatment naïve PD | - |
| Metabolic Cofactor Supplementation in Alzheimer's Disease (AD) and Parkinson's Disease (PD) Patients.  ClinicalTrials.gov Identifier: NCT04044131 |  | Dietary supplementation with cofactors N-acetylcysteine, L-carnitine tartrate, nicotinamide riboside and serine may enhance hepatic β-oxidation resulting in increased mitochondrial activity in the brain. | Double-blind, randomized, placebo-controlled, investigator-initiated, multi-centre trial | Multiple primary outcomes including assessment of cognition, activities of daily living and UPDRS. | 120 participants with PD or Alzheimer’s disease | - |
| 31P-MRS Imaging to Assess the Effects of CNM-Au8 on Impaired Neuronal Redox State in Parkinson's Disease (REPAIR-PD).  ClinicalTrials.gov Identifier:  NCT03815916 |  | CNM-Au8 is a suspension of clean-surfaced, faceted nanocrystalline gold that converts NADH to NAD^+^ to increase intracellular ATP levels, whilst also lowering oxidative stress via superoxide dismutase-like catalytic activity (Soderbom, 2020). | Open-label, investigator-blinded, sequential cohort | Ratio of the oxidized to reduced form of nicotinamide adenine dinucleotide measured by 31P MRS | 24 participants with PD | - |
| Safety and Biomarker Study of EPI-589 in Parkinson's Disease.  ClinicalTrials.gov Identifier: NCT02462603 |  | EPI-589 is a redox active molecule | Phase 2A open-label safety and biomarker study | Safety as assessed by drug-related adverse events | 44 participants – idiopathic and genetic subtype | - |
| Study to Evaluate DNL201 in Subjects With Parkinson's Disease.  ClinicalTrials.gov Identifier:  NCT03710707 |  | Mutations in *LRRK2* are linked to impaired mitochondrial dynamics, bioenergetics, endosomal-lysosomal dysfunction and neuroinflammation (Soderbom, 2020). DNL201 is an orally available, brain-penetrant inhibitor of the LRRK2 | Phase 1b, multicenter, randomized, placebo-controlled, double-blind Study | Safety, tolerability, pharmacokinetics, and pharmacodynamics of DNL201 | 29 participants with PD | - |
| Study to Evaluate DNL151 in Subjects With Parkinson's Disease.  ClinicalTrials.gov Identifier: NCT04056689 |  | See above. DNL151 is also an orally available, brain-penetrant inhibitor of the LRRK2 | Phase 1b, multicenter, randomized, placebo-controlled, double-blind study | Safety, tolerability, pharmacokinetics, and pharmacodynamics of DNL201 | 34 participants with PD | - |
| A Study to Evaluate the Safety, Tolerability, and Pharmacokinetics of BIIB094 in Adults With Parkinson's Disease (REASON).  ClinicalTrials.gov Identifier: NCT03976349 |  | See above. BIIB094 is an antisense oligonucleotide that binds the mRNA for LRRK2 and mediates its degradation | Phase 1 Single- and Multiple-Ascending-Dose Study | Safety, Tolerability, and Pharmacokinetics of BIIB094 | 82 participants with PD | - |
| Ambroxol as a novel disease-modifying treatment for Parkinson's disease dementia.  ClinicalTrials.gov Identifier: NCT02914366 | Protocol published (Silveira et al., 2019) | See above (Mullin et al., 2020) | Phase II, single-centre, double-blind, randomized placebo-controlled trial | Primary outcome measures - Alzheimer's disease Assessment Scale-cognitive subscale and the ADCS Clinician's Global Impression of Change | 75 individuals with mild to moderate PD dementia | - |

CoQ10, coenzyme Q10; CSF, cerebrospinal fluid; MDS, Movement Disorder Society; MRS, magnetic resonance spectroscopy; NAD, nicotinamide adenine dinucleotide; NADH, reduced nicotinamide adenine dinucleotide; PD, Parkinson’s disease; SPECT, single-photon emission computed tomography; UDCA, ursodeoxycholic acid; UPDRS, Unified Parkinson’s disease rating scale; 31P, 31phosphorous. ‘Treated’ or treatment in this setting refers to dopaminergic treatment for PD.

# Additional unpublished clinical trials were identified using the search terms “Parkinson disease” and “mitochondria” using the ClinicalTrials.gov website (Clinicaltrials.gov. <https://clinicaltrials.gov/> [Accessed September 3, 2020]).

**References**

Athauda, D., Maclagan, K., Skene, S.S., Bajwa-Joseph, M., Letchford, D., Chowdhury, K., Hibbert, S., Budnik, N., Zampedri, L., Dickson, J., Li, Y., Aviles-Olmos, I., Warner, T.T., Limousin, P., Lees, A.J., Greig, N.H., Tebbs, S., and Foltynie, T. (2017). Exenatide once weekly versus placebo in Parkinson's disease: a randomised, double-blind, placebo-controlled trial. *Lancet* 390**,** 1664-1675. doi:10.1016/S0140-6736(17)31585-4

Bender, A., Koch, W., Elstner, M., Schombacher, Y., Bender, J., Moeschl, M., Gekeler, F., Muller-Myhsok, B., Gasser, T., Tatsch, K., and Klopstock, T. (2006). Creatine supplementation in Parkinson disease: a placebo-controlled randomized pilot trial. *Neurology* 67**,** 1262-1264. doi:10.1212/01.wnl.0000238518.34389.12

Crotty, G.F., Ascherio, A., and Schwarzschild, M.A. (2017). Targeting urate to reduce oxidative stress in Parkinson disease. *Exp Neurol* 298**,** 210-224. doi:10.1016/j.expneurol.2017.06.017

El-Hattab, A.W., Zarante, A.M., Almannai, M., and Scaglia, F. (2017). Therapies for mitochondrial diseases and current clinical trials. *Mol Genet Metab* 122**,** 1-9. doi:10.1016/j.ymgme.2017.09.009

Hernandez-Camacho, J.D., Bernier, M., Lopez-Lluch, G., and Navas, P. (2018). Coenzyme Q10 Supplementation in Aging and Disease. *Front Physiol* 9**,** 44. doi:10.3389/fphys.2018.00044

Jenner, P., and Langston, J.W. (2011). Explaining ADAGIO: a critical review of the biological basis for the clinical effects of rasagiline. *Mov Disord* 26**,** 2316-2323. doi:10.1002/mds.23926

Krumpolec, P., Vallova, S., Slobodova, L., Tirpakova, V., Vajda, M., Schon, M., Klepochova, R., Janakova, Z., Straka, I., Sutovsky, S., Turcani, P., Cvecka, J., Valkovic, L., Tsai, C.L., Krssak, M., Valkovic, P., Sedliak, M., Ukropcova, B., and Ukropec, J. (2017). Aerobic-Strength Exercise Improves Metabolism and Clinical State in Parkinson's Disease Patients. *Front Neurol* 8**,** 698. doi:10.3389/fneur.2017.00698

Muller, T., Buttner, T., Gholipour, A.F., and Kuhn, W. (2003). Coenzyme Q10 supplementation provides mild symptomatic benefit in patients with Parkinson's disease. *Neurosci Lett* 341**,** 201-204. doi:10.1016/s0304-3940(03)00185-x

Mullin, S., Smith, L., Lee, K., D'souza, G., Woodgate, P., Elflein, J., Hallqvist, J., Toffoli, M., Streeter, A., Hosking, J., Heywood, W.E., Khengar, R., Campbell, P., Hehir, J., Cable, S., Mills, K., Zetterberg, H., Limousin, P., Libri, V., Foltynie, T., and Schapira, A.H.V. (2020). Ambroxol for the Treatment of Patients With Parkinson Disease With and Without Glucocerebrosidase Gene Mutations: A Nonrandomized, Noncontrolled Trial. *JAMA Neurol* 77**,** 427-434. doi:10.1001/jamaneurol.2019.4611

Olanow, C.W., Rascol, O., Hauser, R., Feigin, P.D., Jankovic, J., Lang, A., Langston, W., Melamed, E., Poewe, W., Stocchi, F., Tolosa, E., and Investigators, A.S. (2009). A double-blind, delayed-start trial of rasagiline in Parkinson's disease. *N Engl J Med* 361**,** 1268-1278. doi:10.1056/NEJMoa0809335

Parkinson Study Group, Q.E.I., Beal, M.F., Oakes, D., Shoulson, I., Henchcliffe, C., Galpern, W.R., Haas, R., Juncos, J.L., Nutt, J.G., Voss, T.S., Ravina, B., Shults, C.M., Helles, K., Snively, V., Lew, M.F., Griebner, B., Watts, A., Gao, S., Pourcher, E., Bond, L., Kompoliti, K., Agarwal, P., Sia, C., Jog, M., Cole, L., Sultana, M., Kurlan, R., Richard, I., Deeley, C., Waters, C.H., Figueroa, A., Arkun, A., Brodsky, M., Ondo, W.G., Hunter, C.B., Jimenez-Shahed, J., Palao, A., Miyasaki, J.M., So, J., Tetrud, J., Reys, L., Smith, K., Singer, C., Blenke, A., Russell, D.S., Cotto, C., Friedman, J.H., Lannon, M., Zhang, L., Drasby, E., Kumar, R., Subramanian, T., Ford, D.S., Grimes, D.A., Cote, D., Conway, J., Siderowf, A.D., Evatt, M.L., Sommerfeld, B., Lieberman, A.N., Okun, M.S., Rodriguez, R.L., Merritt, S., Swartz, C.L., Martin, W.R., King, P., Stover, N., Guthrie, S., Watts, R.L., Ahmed, A., Fernandez, H.H., Winters, A., Mari, Z., Dawson, T.M., Dunlop, B., Feigin, A.S., Shannon, B., Nirenberg, M.J., Ogg, M., Ellias, S.A., Thomas, C.A., Frei, K., Bodis-Wollner, I., Glazman, S., Mayer, T., Hauser, R.A., Pahwa, R., Langhammer, A., Ranawaya, R., Derwent, L., Sethi, K.D., Farrow, B., Prakash, R., Litvan, I., Robinson, A., Sahay, A., Gartner, M., Hinson, V.K., Markind, S., Pelikan, M., et al. (2014). A randomized clinical trial of high-dosage coenzyme Q10 in early Parkinson disease: no evidence of benefit. *JAMA Neurol* 71**,** 543-552. doi:10.1001/jamaneurol.2014.131

Parkinson Study Group, S.-P.D.I.I.I.I. (2020). Isradipine Versus Placebo in Early Parkinson Disease: A Randomized Trial. *Ann Intern Med* 172**,** 591-598. doi:10.7326/M19-2534

Payne, T., Sassani, M., Buckley, E., Moll, S., Anton, A., Appleby, M., Maru, S., Taylor, R., Mcneill, A., Hoggard, N., Mazza, C., Wilkinson, I.D., Jenkins, T., Foltynie, T., and Bandmann, O. (2020). Ursodeoxycholic acid as a novel disease-modifying treatment for Parkinson's disease: protocol for a two-centre, randomised, double-blind, placebo-controlled trial, The 'UP' study. *BMJ Open* 10**,** e038911. doi:10.1136/bmjopen-2020-038911

Silveira, C.R.A., Mackinley, J., Coleman, K., Li, Z., Finger, E., Bartha, R., Morrow, S.A., Wells, J., Borrie, M., Tirona, R.G., Rupar, C.A., Zou, G., Hegele, R.A., Mahuran, D., Macdonald, P., Jenkins, M.E., Jog, M., and Pasternak, S.H. (2019). Ambroxol as a novel disease-modifying treatment for Parkinson's disease dementia: protocol for a single-centre, randomized, double-blind, placebo-controlled trial. *BMC Neurol* 19**,** 20. doi:10.1186/s12883-019-1252-3

Snow, B.J., Rolfe, F.L., Lockhart, M.M., Frampton, C.M., O'sullivan, J.D., Fung, V., Smith, R.A., Murphy, M.P., Taylor, K.M., and Protect Study, G. (2010). A double-blind, placebo-controlled study to assess the mitochondria-targeted antioxidant MitoQ as a disease-modifying therapy in Parkinson's disease. *Mov Disord* 25**,** 1670-1674. doi:10.1002/mds.23148

Soderbom, G. (2020). Status and future directions of clinical trials in Parkinson's disease. *Int Rev Neurobiol* 154**,** 153-188. doi:10.1016/bs.irn.2020.02.009

Storch, A., Jost, W.H., Vieregge, P., Spiegel, J., Greulich, W., Durner, J., Muller, T., Kupsch, A., Henningsen, H., Oertel, W.H., Fuchs, G., Kuhn, W., Niklowitz, P., Koch, R., Herting, B., Reichmann, H., and German Coenzyme, Q.S.G. (2007). Randomized, double-blind, placebo-controlled trial on symptomatic effects of coenzyme Q(10) in Parkinson disease. *Arch Neurol* 64**,** 938-944. doi:10.1001/archneur.64.7.nct60005

Vanitallie, T.B., Nonas, C., Di Rocco, A., Boyar, K., Hyams, K., and Heymsfield, S.B. (2005). Treatment of Parkinson disease with diet-induced hyperketonemia: a feasibility study. *Neurology* 64**,** 728-730. doi:10.1212/01.WNL.0000152046.11390.45

Yoritaka, A., Kawajiri, S., Yamamoto, Y., Nakahara, T., Ando, M., Hashimoto, K., Nagase, M., Saito, Y., and Hattori, N. (2015). Randomized, double-blind, placebo-controlled pilot trial of reduced coenzyme Q10 for Parkinson's disease. *Parkinsonism Relat Disord* 21**,** 911-916. doi:10.1016/j.parkreldis.2015.05.022
